# Solvent Effects in Halogen and Hydrogen Bonding Mediated Electrochemical Anion Sensing in Aqueous Solution and at Interfaces

**DOI:** 10.1002/chem.202101102

**Published:** 2021-05-19

**Authors:** Sophie C. Patrick, Robert Hein, Andrew Docker, Paul D. Beer, Jason J. Davis

**Affiliations:** ^1^ Department of Chemistry University of Oxford South Parks Road Oxford OX1 3QZ UK

**Keywords:** anion recognition, electrochemistry, halogen bonding, host-guest systems, sensors

## Abstract

Sensing anionic species in competitive aqueous media is a well‐recognised challenge to long‐term applications across a multitude of fields. Herein, we report a comprehensive investigation of the electrochemical anion sensing performance of novel halogen bonding (XB) and hydrogen bonding (HB) bis‐ferrocene‐(iodo)triazole receptors in solution and at self‐assembled monolayers (SAMs), in a range of increasingly competitive aqueous organic solvent media (ACN/H_2_O). In solution, the XB sensor notably outperforms the HB sensor, with substantial anion recognition induced cathodic voltammetric responses of the ferrocene/ferrocenium redox couple persisting even in highly competitive aqueous solvent media of 20 % water content. The response to halides, in particular, shows a markedly lower sensitivity to increasing water content associated with a unique halide selectivity at unprecedented levels of solvent polarity. The HB sensor, in contrast, generally displayed a preference towards oxoanions. A significant surface‐enhancement effect was observed for both XB/HB receptive films in all solvent systems, whereby the HB sensor generally displayed larger responses towards oxoanions than its halogen bonding analogue.

## Graphical abstract

**Redox‐active ferrocenyl receptors** enable electrochemical anion sensing in highly competitive aqueous solvent media, with a sensory performance that is markedly dependent on the nature of the non‐covalent interaction. In solution, the halogen bonding sensor (XB) is superior and significantly less sensitive to solvent polarity, while surface immobilisation is associated with an enhanced performance, particularly for the hydrogen bonding (HB) system.

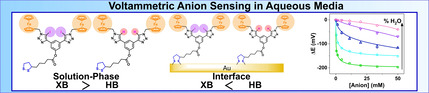

## Introduction

The importance of anions in the environment, medicine and biology necessitates meeting the challenge of their sensing in aqueous media.^[1]^ Owing to an inherently high sensitivity, low‐cost and practical ease, electrochemical anion sensors have received increasing attention, whereby, most commonly, redox‐active anion receptors are interrogated by voltammetric techniques such as cyclic (CV) or square wave voltammetry (SWV).^[2]^ Traditionally, these anion receptors are based on electrostatic or hydrogen bonding (HB) recognition motifs. Recently halogen bonding (XB) has emerged as a particularly useful non‐covalent interaction for anion recognition, especially in aqueous media.^[3]^ Furthermore, XB‐based electrochemical sensors typically display enhanced sensory performances in comparison to HB analogues.^[4]^ Interfacial anion sensing is associated with a number of advantages over solution‐phase sensing, including bypassing host solubility issues, sensor reusability and the potential for a sensing amplification as a result of dielectric effects.[^4f^, ^5^] Despite this, many fundamental sensory properties of these systems remain unexplored and electrochemical anion sensing studies in the presence of significant quantities of water are rare.[^4b^, ^4c^, ^4g^, ^6^] To address this, we herein report a systematic qualitative and quantitative comparison of novel, redox‐active bis‐ferrocene‐(iodo)triazole (**1.XB/HB**) voltammetric sensors towards halides and oxoanions across a range of competitive aqueous organic solvent media. Particular attention is given to resolving the effect of solvent polarity on the magnitude of the sensor response in both solution‐phase and for surface‐bound receptors. This revealed a lower solvent dependence for halide sensing with the XB receptor in solution, underpinning unique selectivity patterns and an enhanced voltammetric response in highly competitive media.

## Results and Discussion

### Synthesis of 1.XB/HB

**Scheme 1:**
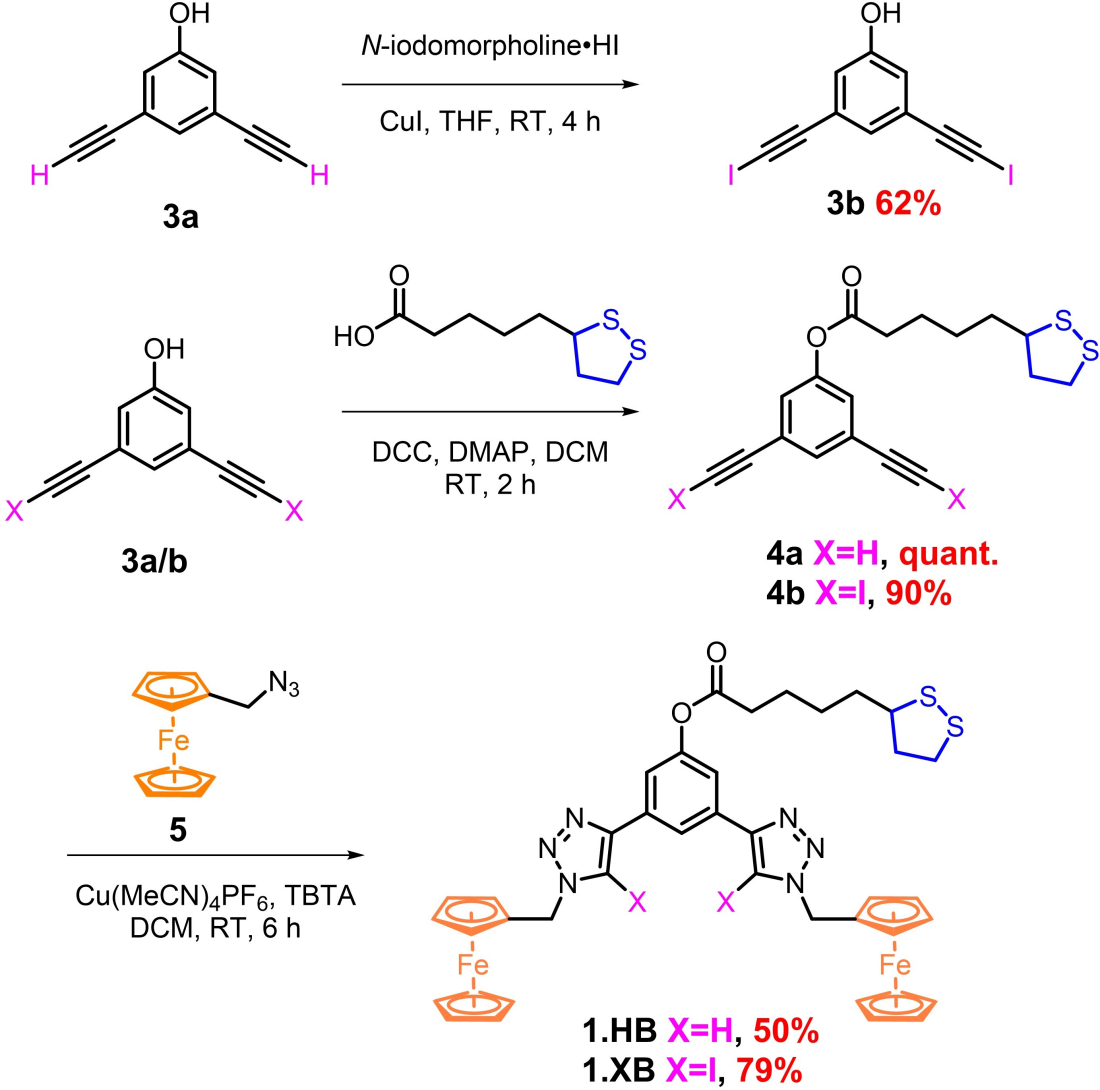
Synthesis of **1.XB/HB**.

The novel receptors **1.XB/HB** were prepared as shown in Scheme 1. The receptor design is based on the 1,3‐benzene‐(iodo)triazole scaffold, a well‐established motif for anion recognition in (aqueous) organic solvent systems,[^3c^, ^3d^, ^4b^, ^6b^, ^7^] and is endowed with a lipoic acid‐derived disulfide anchor group enabling surface immobilisation onto gold electrodes. Appended to each triazole arm of the receptor is a redox‐active ferrocene group. Sonogashira cross‐coupling reaction of 3,5‐dibromo‐phenol with trimethylsilylacetylene, followed by deprotection of the trimethylsilyl (TMS) group afforded **3 a** in moderate yields.^[8]^ This compound was then reacted with N‐iodomorpholine‐hydrogen iodide to afford the bis(iodoalkyne) **3 b**. Esterification of **3 a/b** with lipoic acid was carried out using dicyclohexylcarbodiimide and 4‐dimethylaminopyridine (DCC‐DMAP) to give the disulfide‐appended bis‐alkynes **4 a/b** in good yields. Copper(I)‐catalysed alkyne‐azide cycloaddition (“click” reaction) of these synthons with two equivalents of azide methylene functionalised ferrocene **5** ^[9]^ afforded the target receptors **1.XB/HB**. Further experimental details and NMR, mass spectroscopic characterisation of the receptors is detailed in the Supporting Information (S2).

### Electrochemical characterisation

**Figure 1:**
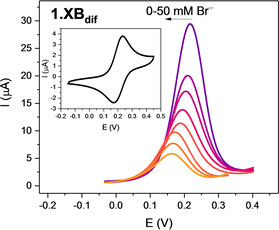
Evolution of square‐wave voltammograms (SWVs) of 0.1 mM **1.XB_dif_** in ACN (100 mM TBAClO_4_) upon titration of TBABr up to 50 mM. The inset shows the corresponding CV at a scan rate of 0.1 V s^−1^. Potentials are reported with respect to Ag|AgNO_3_.

In all cases a well‐defined single‐electron redox wave attributed to the Fc/Fc^+^ couple was observed for both receptors under diffusive conditions (denoted **1.XB/HB_dif_**), as representatively shown for ACN, 100 mM TBAClO_4_ in Figure 1 and Figure S11. In this solvent a difference in the half‐wave potential E_1/2_ of 12 mV was observed between the XB and HB analogues, (219±1 mV and 205±2 mV vs. Ag|AgNO_3_, respectively), attributed to the greater electron‐withdrawing ability of the iodo‐triazole vs. proto‐triazole analogue.[^4a^, ^4b^, ^4c^] Of particular note is that in all solvents (ACN and ACN/water mixtures) only one oxidation wave was observed indicating that both Fc groups were addressed simultaneously (no electronic communication; see also the square‐wave voltammograms (SWV) in Figure S12). Quasi‐reversibility of the redox couple was ascertained by virtue of varying the voltammetric scan rate, as discussed in more detail in the Supporting Information (Figures S13–S15).

### Solution‐phase anion sensing

**Figure 2:**
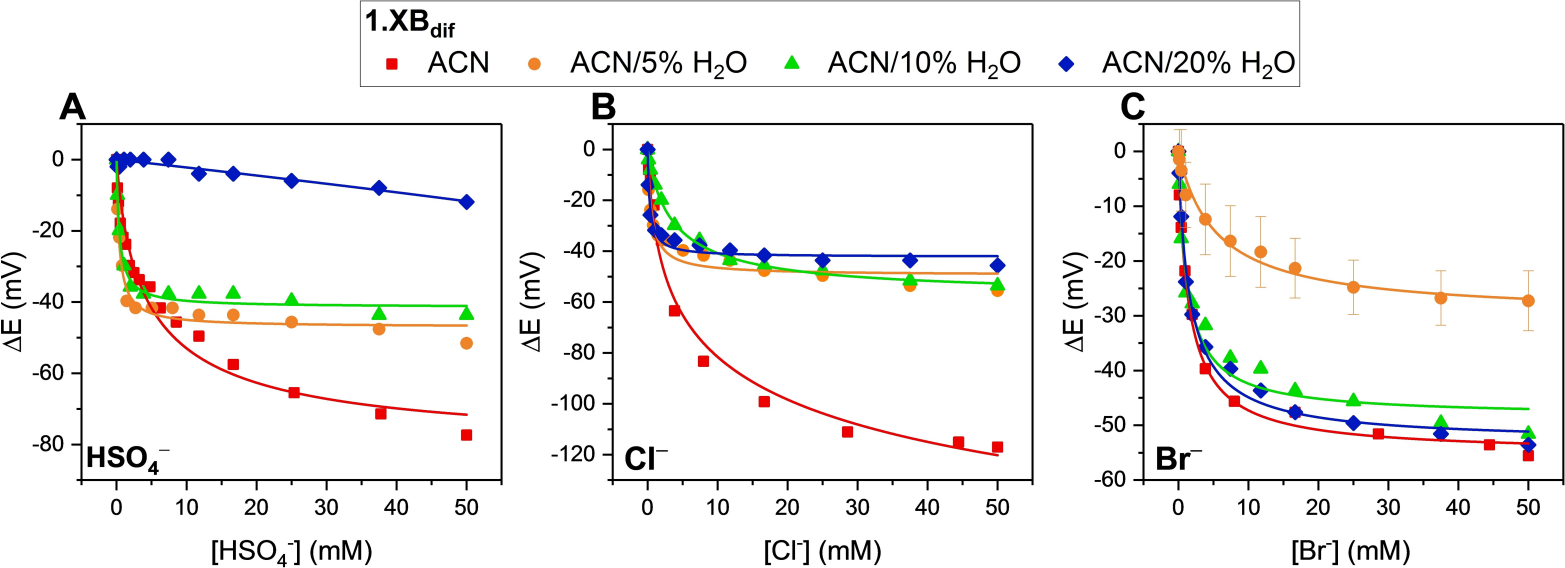
Cathodic voltammetric shifts of **1.XB_dif_** in ACN with varying water content ([**1.XB_dif_**]=0.1 mM with 100 mM TBAClO_4_ supporting electrolyte), upon titration of A) HSO_4_^−^ B) Cl^−^ and C) Br^−^. The overall ionic strength was kept constant at 100 mM throughout. Solid lines represent fits according to the 1 : 1 host‐guest Nernst model (Equation (2)). The equivalent isotherms for **1.HB_dif_** are shown in Figure S20. A direct comparison for the response of **1.XB/HB_dif_** towards these anions (HSO_4_^−^, Cl^−^ and Br^−^) as well as NO_3_^−^ and H_2_PO_4_^−^ across all solvent systems is also shown in the Supporting Information (Section S3, Figures S21 and S22). Errors in C) for Br^−^ in ACN/5 % H_2_O represent one standard deviation of two independent measurements.^[12]^

Electrochemical voltammetric solution‐phase anion sensing studies were carried out by monitoring the receptors Fc/Fc^+^ E_1/2_ (by SWV) upon titration with various anions (at constant ionic strength; Figures 1 and 2 and S16, Table 1). Substantial cathodic shifts were observed for both **1.XB/HB_dif_** in ACN of up to −120 mV with **1.XB_dif_** in the presence of Cl^−^, (Figures 2 and S16 A), while, in this solvent H_2_PO_4_^−^ also induced large perturbations, but affected reversibility to such a degree that quantitative analysis was not possible. As shown in Table 1 and Figure S16A, in ACN **1.XB_dif_** displayed the following selectivity trend in terms of maximum cathodic shift ΔE_max_: Cl^−^>HSO_4_^−^>Br^−^>NO_3_^−^, while **1.HB_dif_** displayed a response preference towards HSO_4_^−^>Cl^−^≈Br^−^>NO_3_^−^ and notably a strongly attenuated response towards chloride of −38 mV. These trends are similar to those recently reported for Fc‐isophthalamide‐(iodo)triazole receptors **2.XB/HB_dif_** (see Supporting Information for structures and further comparisons, Section S3, Figure S17).^[4f]^ It is noteworthy that **1.XB/HB_dif_** display a generally larger response magnitude (Figures S18 and S19), potentially as a result of a larger anion binding switch‐on upon oxidation by virtue of the presence of two appended Fc‐transducers. The specific effects of this are currently under investigation.^[10]^

**Table 1:** Cathodic shift ΔE_max_ (mV) of **1.XB/HB_dif_** in a range of ACN/H_2_O mixtures in the presence of 50 mM of various anions. Estimated error ±5 mV. / – Investigations restricted by poor redox reversibility. n/a – not conducted.

ΔE_max_ [mV]								
	ACN		ACN/5 % H_2_O		ACN/10 % H_2_O		ACN/20 % H_2_O	
	**XB**	**HB**	**XB**	**HB**	**XB**	**HB**	**XB**	**HB**
HSO_4_^−^	−77	−79	−52	−40	−44	−26	−12	−8
H_2_PO_4_^−^	/	/	−133	−129	−60	−62	−41	−32
NO_3_^−^	−39	−16	−15	−5	n/a	n/a	n/a	n/a
Cl^−^	−120	−38	−56	−18	−54	−20	−46	0
Br^−^	−56	−34	−27±5	−19±1	−52	−28	−54	−6

As observed for other XB redox active receptors,[^4a^, ^4b^, ^4c^, ^4f^] the magnitude of response of **1.XB_dif_** almost always exceeded that of **1.HB_dif_**, in both ACN as well as more competitive aqueous organic solvent systems (vide infra).

The sensing capabilities of **1.XB/HB_dif_** were then investigated in more competitive media (ACN with increasing water content) where substantial cathodic perturbations were still observed even in the highly competitive ACN/20 % H_2_O (Table 1, Figures 2 and S16D).^[11]^ For both sensors the relative responses for the oxoanions was H_2_PO_4_^−^>HSO_4_^−^>NO_3_^−^ across all solvent systems. The HB sensor retained a preference towards the oxoanions H_2_PO_4_^−^ and HSO_4_^−^ over halides in all solvents. In stark contrast, in competitive ACN/20 % H_2_O, **1.XB_dif_** gave the greater responses to the halides, highlighting an important and unique selectivity switching for the XB sensor.

**Figure 3:**
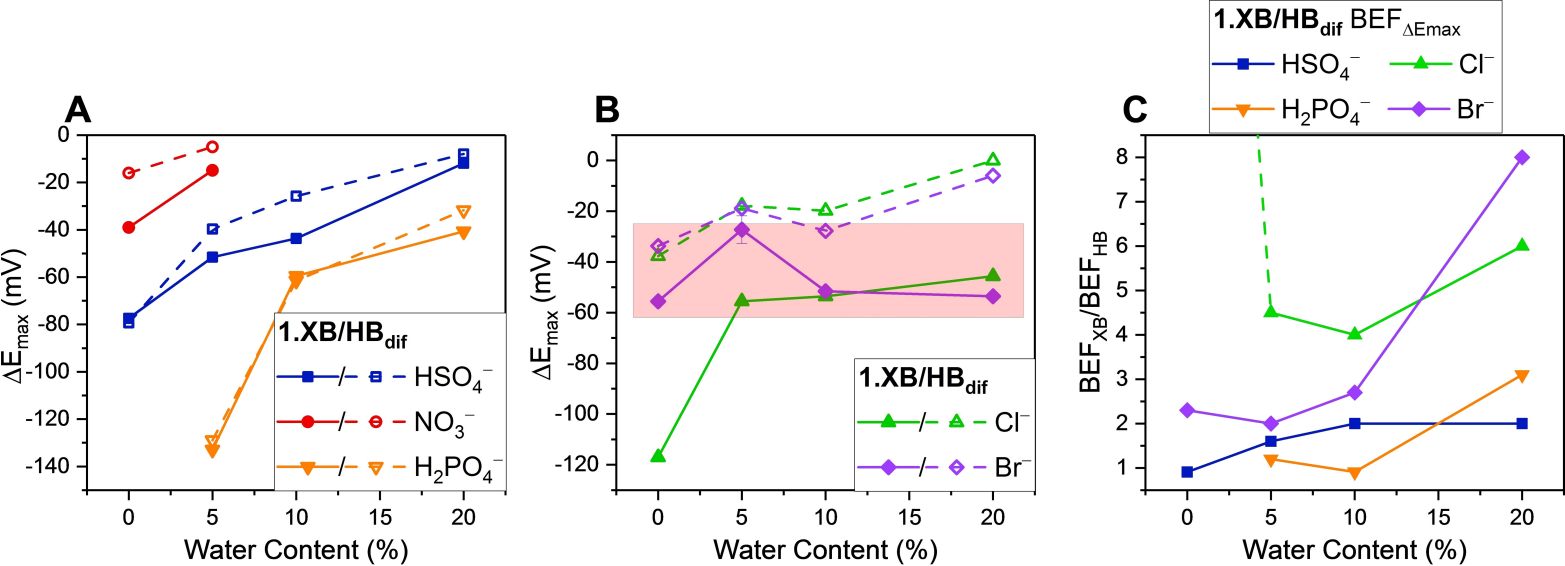
Maximum cathodic perturbation of E_1/2_ (ΔE_max_) of **1.XB_dif_** (filled symbols) and **1.HB_dif_** (empty symbols) in a range of solvent systems with varying water content, in the presence of 50 mM of A) the oxoanions (HSO_4_^−^, NO_3_^−^ and H_2_PO_4_^−^), and B) halides (Cl^−^ and Br^−^) with connecting lines to guide the eye only. Note that the axis scaling for both graphs is identical. Errors in B) for Br^−^ in ACN/5 % H_2_O represent one standard deviation of two independent measurements. The red box in B) highlights the region wherein the sensor performance of **1.XB_dif_** is largely unaffected by water content. A quantitative comparison of the slopes of each ΔE_max_ vs. water content plot is detailed in the Supporting Information (Section S4, Figure S23 and S24, Table S1). C) Ratios of BEFs for **1.XB_dif_** (BEF_XB_) and **1.HB_dif_** (BEF_HB_), i. e. the XB enhancement factors, under diffusive conditions in ACN/H_2_O mixtures of different water content. The BEFs were obtained from ΔE_max_ and Equation (1). The analogous analysis via fitting of the isotherms according to Equation (2) affords largely identical values and trends, as shown in Figures S26 and S27. The XB enhancement factor for Cl^−^ in ACN is 30 and is omitted for scaling reasons (indicated by dashed green line).

Table 1 shows that, as expected, the maximum cathodic shift for each anion (at 50 mM) generally decreases as the water content of the solvent system increases (Figures 2 and 3 and S21 and S22). There are however some notable differences in the specific trends. The maximum response magnitude towards the oxoanions was similar for **1.XB_dif_** and **1.HB_dif_** and also diminished comparably for both upon the introduction of water (Figure 3A–B). Specifically, the response of both **1.XB_dif_** and **1.HB_dif_** towards HSO_4_^−^ decreased by ≈3.2 mV/%H_2_O, while the sensor response towards H_2_PO_4_^−^ was even more sensitive towards an increased polarity (≈5.8 mV/%H_2_O), observations consistent with the large hydration enthalpy of this anion (Table S1, Figures S23 and S24).

In contrast, the halides displayed more nuanced trends. Firstly, the difference in sensing performance between **1.XB_dif_** and **1.HB_dif_** is more pronounced for these anions across all solvents, with the former generally displaying a significantly larger response. Furthermore, the XB motif displayed a significantly smaller solvent dependence in terms of response magnitude towards both Cl^−^ and Br^−^. A noteworthy exception is the initially large response drop of **1.XB_dif_** towards Cl^−^ from ACN (−120 mV) to ACN/5 % H_2_O (−56 mV), while the addition of even more water did not significantly affect the sensor response (−54 mV in ACN/10 % H_2_O, see Figure 2B). This behaviour is most likely related to unique hydration effects and warrants further attention in future studies.

A lowered solvent polarity dependence is particularly noticable for Br^−^ at **1.XB_dif_**, whose response is nearly constant, regardless of solvent polarity, as highlighted in Figure 3B with a near‐zero slope for ΔE_max_ vs. water content (the response of **1.HB_dif_** decreased by 1.2 mV/%H_2_O).^[12]^ Similarly, the HB sensors' response towards Cl^−^ dropped by 1.3 mV/%H_2_O, while **1.XB_dif_** was only half as sensitive to an increased solvent polarity with 0.7 mV/%H_2_O (disregarding the first data point in ACN). The *relative* halide sensing performance of the XB sensors thus increases as solvent water content grows. This is a significant unprecedented observation, consistent with a previously reported lower dependence of XB on solvent polarity.^[13]^

The combined observations of an enhanced voltammetric XB sensor response together with a lower solvent dependence saliently highlights the enormous potential of XB based platforms as anion receptors and sensors in aqueous solvents.[^3a^, ^3b^, ^3f^, ^4g^] Herein, this is reflected in a significant sensing performance of **1.XB_dif_** in the highly competitive ACN/20 % H_2_O, with cathodic perturbations of up to −54 mV for Br^−^. To the best of our knowledge this is the most competitive aqueous solvent system for which XB mediated voltammetric anion sensing has been reported and has been achieved with relatively simple, natively charge neutral receptors.[^4b^, ^4c^]

We further quantified the solvent dependencies of both sensors through quantitative analysis of the electrochemical titration binding isotherms. The magnitude of the voltammetric shift ΔE is in its most general form given by Equation (1) and is primarily determined by the ratio of the anion binding constants to the different receptor oxidation states K_Ox_/K_Red_, often denoted as the binding enhancement factor (BEF).^[14]^

(1) $$\Delta E=-\frac{RT}{nF}\ln\left(\frac{K_{Ox}}{K_{Red}}\right)$$

From ΔE_max_ (as shown in Tables 1 and S2 and Figure 3), the BEFs were directly obtained via Equation (1) and are collated in Table S3. As expected, these BEFs generally display the same trends as shown for ΔE_max_ in Figure 3A–B. We can, additionally, resolve the XB enhancement factor (XBEF), the ratio of the BEFs of the XB and HB systems (XBEF=BEF_XB_/BEF_HB_). This provides a quantitative measure of the superior XB sensor performance, in terms of its response to anionic guests, with respect to its HB analogue.

In excellent agreement with the enhanced solution‐phase sensing capability of **1.XB_dif_** over **1.HB_dif_** are the significant XB enhancement factors (>1) for almost all anions in all solvent systems, as depicted in Figures 3C and S25. Not only are these enhancements surprisingly large, in some cases (e. g. XBEF=30 for Cl^−^ in ACN and XBEF=8 for Br^−^ in ACN/20 % H_2_O), but also quantitatively corroborate the trends discussed above. Firstly, a clear relative response preference of the XB receptor towards the halides over the oxoanions is ascertained. Secondly, and very importantly, an increased XB enhancement is observed upon increasing water content of the solvent for all anions which may be attributed to the unique XB‐anion bonding interaction, containing a significant covalent bonding contribution.^[15]^

In order to determine absolute values for K_Ox_ or K_Red_ an extended model, Equation (2), which is applicable under fast‐exchange, continuous shift conditions and when [A^**−**^]≫[H] (where [A^**−**^] and [H] are the concentrations of the anion and host, respectively) is used (Table S3–S7.[^4e^, ^16^]

(2) $$\Delta E=-\frac{RT}{nF}\ln\left(\frac{1+K_{Ox}\left[A^{-}\right]}{1+K_{Red}\left[A^{-}\right]}\right)\;$$

As shown in Figures 2, S16 and S21, this extended model affords good fits to the experimental isotherms, indicating that one anion binds to the (dicationic) receptors. As expected, in most cases the absolute binding constants obtained decrease upon increasing the competitiveness/polarity of the solvent (Tables S3–S7), with BEFs pleasingly of similar magnitude to those determined from Equation (1) and ΔE_max_ (see Figures S26 and S27 for a direct comparison).

### Surface immobilisation and characterisation

**Figure 4:**
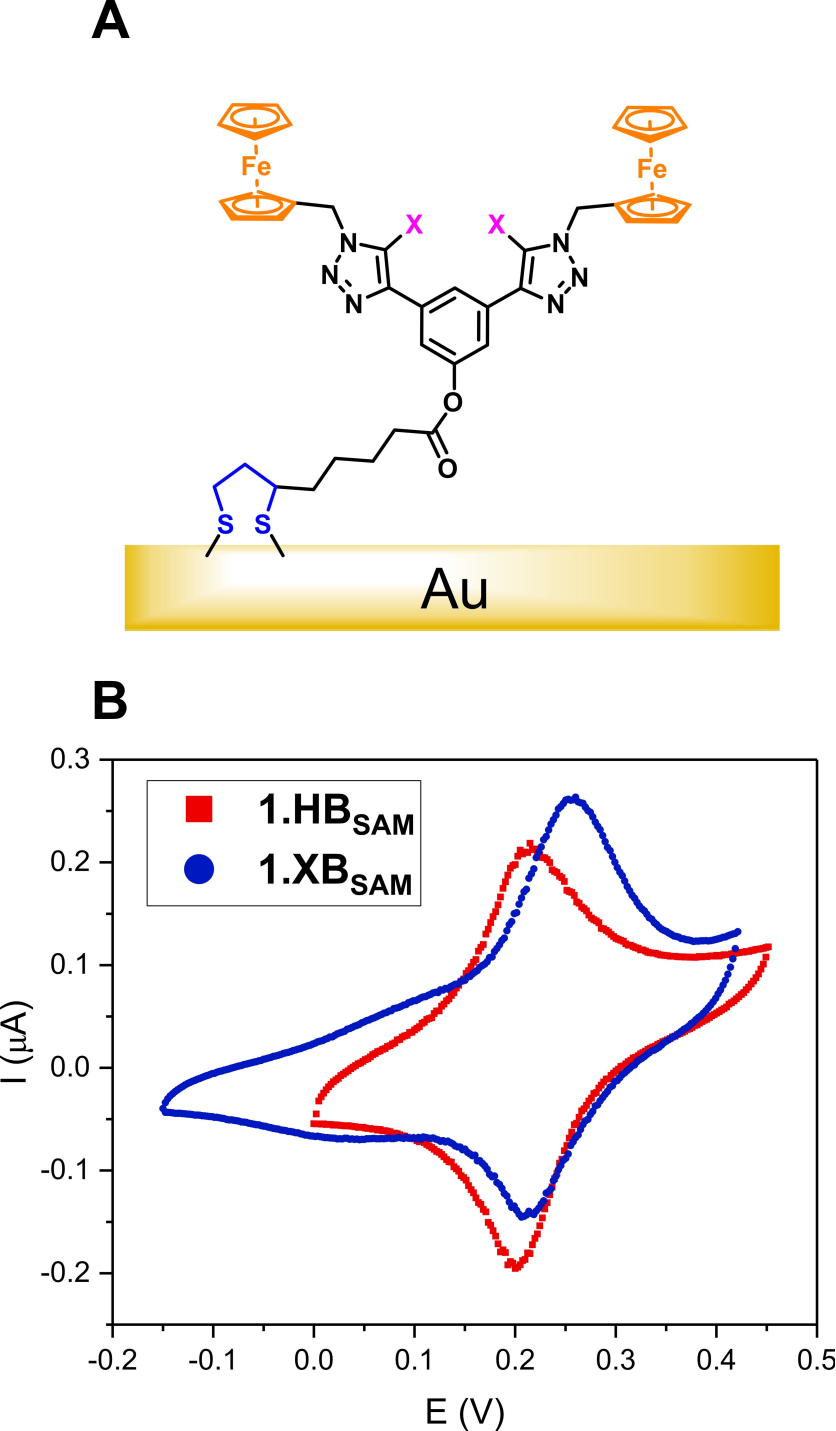
A) Schematic representation of **1.XB/HB_SAM_** films immobilised on a gold surface and B) cyclic voltammograms of **1.XB/HB_SAM_** in ACN, 100 mM TBAClO_4_, at a scan rate of 100 mV s^−1^. Potentials are reported with respect to Ag|AgNO_3_.

Formation of self‐assembled monolayers (SAMs) of the receptors was achieved by incubating clean Au electrodes in a solution of 1 mM **1.XB/HB** in DCM overnight to enable chemisorption of the disulfide (**1.XB/HB_SAM_**, Figure 4A). High water contact angles of **1.XB/HB_SAM_** (77±2° vs. 86±1°, respectively, Table 2) suggested that, as expected, hydrophobic molecular films arise.^[17]^

**Table 2:** Surface characterisation data of **1.XB/HB_SAM_**, including water contact angle measurements, ellipsometric SAM thickness, d, molecular surface coverage, Γ, E_1/2_ in ACN (100 mM TBAClO_4_) vs. Ag|AgNO_3_, dielectric constant, *ϵ*_r_, and capacitance, C.

	Water contact angle [°]^[a]^	d [nm]^[b]^	Γ [10^−11^ mol cm^−2^]^[a]^	Molecular footprint [nm^2^]^[a]^	E_1/2_ [mV]^[a]^	*ϵ*_r_^[a]^	C [μF cm^−2^]^[a]^
**1.XB_SAM_**	77±2	1.45±0.50	6.2±1.2	2.8±0.6	222±3	12±0.7	7.3±0.4
**1.HB_SAM_**	86±1	0.95±0.3	8.4±0.2	2.0±0.1	212±1	4±0.8	3.4±0.7

Errors represent one standard deviation of [a] independent experiments on 3 electrodes or [b] 3 repeat measurements on one substrate.

Successful SAM formation was further evidenced by attenuated total reflection‐Fourier transform infrared (ATR‐FTIR) measurements which revealed matching spectra for both the receptors and their SAMs (see Figure S28 and Section S5). Film thicknesses d of 1.45±0.50 and 0.95±0.3 nm, determined *via* ellipsometry, are consistent with monolayer formation and potentially indicative of molecular conformations that are tilted towards the Au surface to some extent and molecular conformations that are slightly different for the XB and HB motifs (see Supporting Information Section S5.1 for further discussions).^[18]^ A molecular tilt is further supported by consideration of the molecular surface coverages *Γ* (determined from charge integration of the Fc peaks in CV), which are with 6.2±1.2 and 8.4±0.2 mol cm^−2^ (corresponding to molecular footprints of 2.8±0.6 and 2.0±0.1 nm^2^), for **1.XB/HB_SAM_**, respectively, slightly lower than those of similar receptive SAMs.[^4f^, ^4g^]

Film thicknesses were then used in conjunction with resolved capacitances of 7.3±0.4 and 3.4±0.7 μF cm^−2^, respectively (determined by impedance‐derived capacitance spectroscopy), to calculate film dielectric constants ϵ from a Helmholtz model, affording values of 12±0.7 vs. 4±0.8 for **1.XB/HB_SAM_**, respectively (see Supporting Information S5.1 for further details).

The significantly lower ϵ for **1.HB_SAM_** might arise from a denser packing of the SAM (also resolved faradaically) and a concomitantly reduced solvent penetration and is relevant to the film response changes with solvent (see below). The voltammetric properties of **1.XB/HB_SAM_** were in good agreement with their solution‐phase counterparts with well‐defined, redox traces observed by CV (Figure 4) and SWV (Figure S12), and a notable difference of 10 mV between the E_1/2_ of **1.XB/HB_SAM_** analogues (212±1 mV and 222±3 mV in ACN, Table 2). As expected for a surface‐immobilised redox couple, a good linear relationship was obtained for plots of the peak currents i_p_ vs. the scan rate υ (Figures S29 and S30).

### Interfacial anion sensing

**Figure 5:**
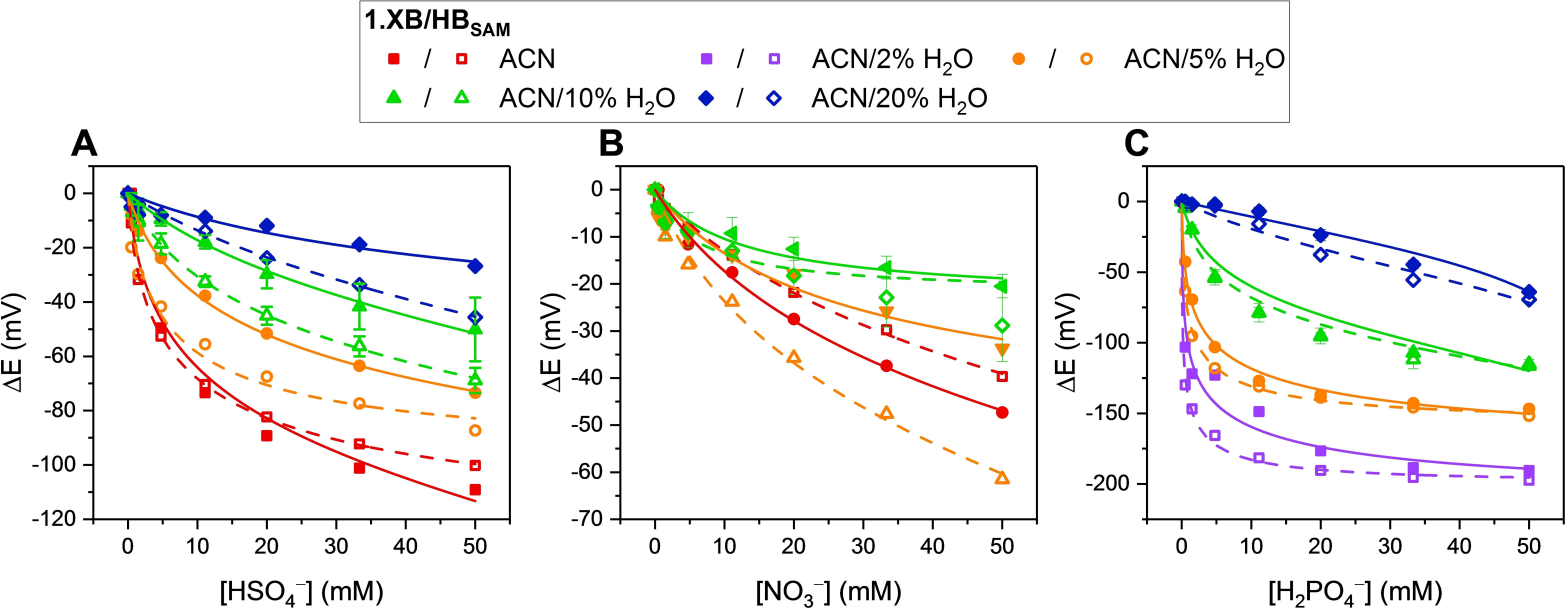
Cathodic voltammetric shifts of **1.XB_SAM_** (filled symbols) and **1.HB_SAM_** (empty symbols) in a range of ACN solvent systems with varying water content. Response of **1.XB/HB_SAM_** upon titration of 50 mM A) HSO_4_^−^ B) NO_3_^−^ and C) H_2_PO_4_^−^. The overall ionic strength was kept constant at 100 mM throughout. Solid lines represent fits according to the 1 : 1 host‐guest Nernst model (Equation (2)). Error bars, where shown, represent one standard deviation of three independent measurements.

Interfacial anion sensing studies were conducted across similar solvent systems as investigated diffusively (ACN and with 1, 2, 5, 10, 20, 30 % H_2_O, 100 mM TBAClO_4_). As a result of poor redox reversibility and/or overlapping redox activity, no systematic studies could be carried out with halides. However, a comprehensive comparison of performance towards oxoanions was conducted. A consistent trend was observed where H_2_PO_4_^−^ elicited the largest response for both SAMs across all solvent systems (up to −200 mV for **1.HB_SAM_** in ACN/2 % H_2_O) followed by HSO_4_^−^, then NO_3_^−^ (Figures 5, S31 and S32, Table 3). In ACN, **1.XB_SAM_** outperforms its HB analogue in the presence of both HSO_4_^−^ (−110 vs. −100 mV) and NO_3_^−^ (−47 vs. −40 mV). Interestingly, a reversal in performance is observed in the presence of water (even 1 % H_2_O), where **1.HB_SAM_** consistently displays larger maximum cathodic perturbations than **1.XB_SAM_** in response to all the anions (Table 3).

**Table 3:** Cathodic shift ΔE_max_ (mV) of **1.XB/HB_SAM_** in a range of ACN/H_2_O mixtures in the presence of 50 mM of various anions. Estimated error ±5 mV. / – Investigations restricted by poor redox reversibility or overlapping redox potentials with anion. Titrations of Cl^−^ in ACN/10 % H_2_O displayed maximum cathodic perturbations of −34 and −49 mV for **1.XB/HB_SAM_** respectively, but studies in all other solvent systems were restricted (see Supporting Information for more information, Section S5). n/a – not conducted.

ΔE_max_ [mV]														
	ACN		ACN/1 % H_2_O		ACN/2 % H_2_O		ACN/5 % H_2_O		ACN/10 % H_2_O		ACN/20 % H_2_O		ACN/30 % H_2_O	
	**XB**	**HB**	**XB**	**HB**	**XB**	**HB**	**XB**	**HB**	**XB**	**HB**	**XB**	**HB**	**XB**	**HB**
HSO_4_^−^	−110	−100	−81	−120	−89	−110	−73	−87	−50	−69	−27	−46	−26	−34
H_2_PO_4_^−^	/	/	/	/	−190	−200	−150	−150	−120	−120	−64	−76	−24	−40
NO_3_^−^	−47	−40	−62	−60	−26	−67	−34	−62	−21	−29	n/a	n/a	n/a	n/a

**Figure 6:**
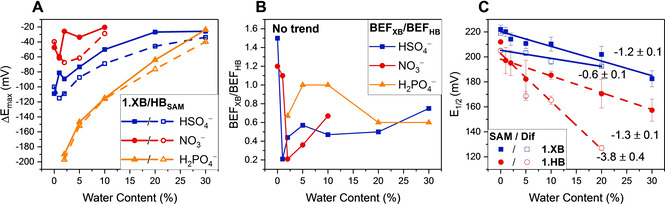
A) Maximum cathodic perturbation of E_1/2_ (ΔE_max_) of **1.XB/HB_SAM_** in a range of ACN solvent systems with varying water content, in response to 50 mM HSO_4_^−^, NO_3_^−^ and H_2_PO_4_^−^. B) The XB enhancement factor given by BEF_XB_/BEF_HB_ for **1.XB/HB_SAM_**. A direct comparison across all solvent systems is also shown in the Supporting Information (Section S4, Figure S21). C) E_1/2_ of **1.XB/HB_dif/SAM_** vs. percentage of H_2_O in ACN (v/v) including linear fits with the magnitude of the slopes shown in mV/%. Error bars represent one standard deviation of at least 3 independent measurements.

However, in contrast to solution‐phase conditions, the difference in sensing performance between both receptive films is relatively smaller and in almost all cases **1.HB_SAM_** outperforms **1.XB_SAM_** i. e., the XB enhancement trends observed in solution are reversed for the SAMs (Figure 6B) where **1.HB_SAM_** outperforms the XB interface (i. e. BEF_XB_/BEF_HB_ <1) regardless of solvent composition.

As the water content of the solvent media increased above ACN/10 % H_2_O, a deviation from the typical response isotherm was observed for HSO_4_^−^ and H_2_PO_4_^−^, the origins of which are currently under investigation. As expected, the magnitude of maximum cathodic shift for each oxoanion generally decreased as the water content of the solvent system increased (Figures 5 and 6A and S33).

Interestingly, this dependence was not only equal for both films but also largely identical to that observed under diffusive conditions with ≈2.7 and ≈5.4 mV/%H_2_O for HSO_4_^−^ and H_2_PO_4_^−^, respectively (Table S1).

The improved SAM performance, with a significant response in the highly competitive ACN/30 % H_2_O system (*e. g*. −40 mV for **1.HB_SAM_** towards H_2_PO_4_^−^) thus arises as a result of an enhanced response magnitude through surface immobilization (i. e. a larger “baseline” response), and not an altered solvent dependence. The increased general response of the SAMs compared to solution can be defined through a surface enhancement factor (SEF) obtained via: SEF=BEF_SAM_/BEF_dif_ (Figure S34, Tables S11–S13). In all cases the SEF is ≥1 and can be over one order of magnitude.^[4f]^

A quantitative analysis of the interfacial sensing performance of **1.XB/HB_SAM_** was undertaken by fitting to the 1 : 1 host‐guest Nernst model (Equation (2)). Good fits were obtained for all isotherms in solvent systems with ≤10 % H_2_O, as discussed in the Supporting Information (Sections S5.3 and S6.3, Figures S31 and S32 A, Tables S8–S10). In the presence of higher concentrations of water a deviation from this model was observed (Figures S32B and S32 C), the origins of which are currently under investigation.

### Further comparison of diffusive and interfacial sensing performance

As highlighted throughout, a prominent surface enhancement effect was observed across sensing studies in all solvents, with the magnitude of the (maximum) cathodic shifts of **1.XB/HB_SAM_** often being several times larger than that of **1.XB/HB_dif_** (Figures S23 and S24). We have recently proposed a new model that can account for this observation based on a consideration of the SAM dielectric constant.^[4f]^ Specifically, the larger interfacial response magnitude is reflective of a larger interfacial BEF, attributable to a promoted K_Ox_. This enhanced K_Ox_ arises from diminished through‐space charge screening between Fc^+^ and anion in the low‐dielectric SAM. This is directly supported here by consideration of the measured film dielectric constants ϵ of 12±0.7 and 4±0.8 for **1.XB/HB_SAM_**, respectively (vide supra). The higher dielectric of the XB interface is, based on the above‐mentioned principles, in good agreement with the comparably diminished interfacial response of **1.XB_SAM_** over **1.HB_SAM_** and can thus account for the counterintuitively superior interfacial HB sensing performance. The unexpectedly large differences in film dielectric are likely to originate from differences in film conformation or hydration (see Supporting Information Section S5), a proposal which is broadly consistent with the relative molecular densities referred to above.

It should also be noted that the unexpectedly improved performance of the **1.HB_SAM_** is, in part, also a reflection of a relatively inferior solution‐phase performance of **1.HB_dif_**. One potential origin of this would be a greater interaction of this motif with polar solvent.

A mapping of receptor E_1/2_ as a function of water content is in direct support of this (Figure 6C). In all cases an increased solvent polarity translates to a stabilisation of the Fc^+^ redox state and thus a lower E_1/2_. For the XB motifs and **1.HB_SAM_** and **1.XB_dif_** this dependence is the same within error. For the **1.HB_dif_** motif, however, there is a markedly greater response to solvent, suggestive of the same increased polar solvent interaction that diminishes sensor performance.

## Conclusion

Novel XB and HB disulfide‐appended bis(ferrocene‐(iodo)triazole) redox‐active anion receptors **1.XB/HB** were prepared and subjected to comprehensive diffusive and interfacial anion sensing studies across a wide range of organic‐aqueous solvent mixtures (ACN/H_2_O). In solution, the XB sensor displayed significantly larger cathodic responses to a range of oxoanions and halides over the analogous HB receptor. Significantly, this XB enhancement not only persisted across all solvent systems, but relatively increased in the presence of higher concentrations of water (in particular for the halides). This lower XB solvent dependence is likely to be a reflection of an enhanced degree of covalency and/or an altered receptor solvation. Importantly, it not only enables sensing in highly competitive aqueous solvent media but is also associated with a unique halide selectivity.

Upon immobilisation of these receptors within SAMs a significant enhancement in response was observed in line with prior reports. Unexpectedly, the HB interface now, in general, outperformed the XB interface sensor, most likely as a result of local dielectric effects reflective of film organisational or hydration differences. Both receptive SAMs were similarly sensitive to increasing water content, with an absolute decrease in oxoanion sensing performance comparable to that observed in solution.

The promising results presented herein serve to further improve our fundamental understanding of diffusive and interfacial electrochemical XB and HB anion sensing, potentially leading to the development of sensory devices capable of functioning under aqueous conditions.

## Experimental Section

General information as well as further details about compound characterisation, surface analyses and sensing protocols are detailed in the Supporting Information.

**Synthesis of 3 b**: 3,5‐diethynyl phenol **3 a** (500 mg, 3.52 mmol) (synthesised according to literature [8]), N‐Iodomorpholine hydriodide (3.61 g, 10.6 mmol) and CuI (67 mg, 0.352 mmol) were dissolved in anhydrous THF (tetrahydrofuran) (22 ml) and left to stir excluded from the presence of light for 4 h at room temperature. Afterwards the reaction mixture was diluted with CH_2_Cl_2_ (100 ml) and filtered through a CH_2_Cl_2_ saturated alumina pad. The organic phase subsequently washed with 0.01 M NH_4_OH/EDTA (ethylenediaminetetraacetic acid) solution, dried over MgSO_4_ filtered and solvent removed in vacuo. The crude material was subjected to silica‐gel column chromatography and isolated as a white solid (792 mg, 2.01 mmol, 57 %). ^1^H NMR (400 MHz, CDCl_3_) δ 7.08 (t, *J*=1.4 Hz, 1H_c_), 6.86 (d, *J*=1.4 Hz, 2H_b_), 5.22 (s, 1H_a_). ^13^C NMR (101 MHz, CDCl_3_) δ 155.19, 129.13, 124.84, 119.92, 92.91, 7.95. HRMS (ESI‐ve) m/z: 392.82748 ([M−H]^−^, C_10_H_3_O^127^I_2_ requires 392.82787).

**Synthesis of 4 a**: 3,5‐diethynyl phenol **3 b** (100 mg, 0.704 mmol), lipoic acid (174 mg, 0.845 mmol), dicyclohexylcarbodiimide (192 mg, 0.930 mmol) and ca. 5 mg of 4‐dimethylaminopyridine (DMAP) were dissolved in anhydrous CH_2_Cl_2_ (20 ml) and left to stir at room temperature. After 2 hours the mixture was diluted with CH_2_Cl_2_ (10 ml) filtered and the resultant filtrate was concentrated in vacuo. The crude residue was subjected to silica‐gel column chromatography and isolated as a yellow solid (233 mg, 0.704 mmol, quantitative). ^**1**^ **H** NMR (500 MHz, CDCl_3_) δ 7.46 (s, 1H_i_), 7.23–7.18 (m, 2H_h_), 3.65–3.53 (m, 1H_e_), 3.25–3.03 (m, 4H_g,j_), 2.57 (t, *J*=7.5 Hz, 2H_a_), 2.52–2.42 (m, 1H_f_), 2.00–1.85 (m, 1H_f_), 1.84–1.66 (m, 4H_b,d_), 1.64–1.50 (m, 2H_c_). ^13^C NMR (126 MHz, CDCl_3_) δ 171.49, 150.34, 133.22, 125.94, 123.81, 81.78, 78.92, 56.37, 40.36, 38.65, 34.70, 34.13, 28.78, 24.67. HRMS (ESI+ve) m/z: 331.08228 ([M+H]^+^, C_18_H_19_O_2_^32^S_2_ requires 331.08210).

**Synthesis of 4 b**: **3 b** (200 mg, 0.508 mmol), lipoic acid (126 mg, 0.609 mmol), dicyclohexylcarbodiimide (115 mg, 0.558 mmol) and ca. 5 mg of DMAP were dissolved in anhydrous CH_2_Cl_2_ (20 ml) and left to stir at room temperature. After 2 hours the mixture was diluted with CH_2_Cl_2_ (10 ml) filtered and the resultant filtrate was concentrated in vacuo. The crude residue was subjected to silica‐gel column chromatography and isolated as a yellow solid (266 mg, 0.457 mmol, 90 %). ^1^H NMR (400 MHz, CDCl_3_) δ 7.35 (t, *J*=1.4 Hz, 1H_i_), 7.12 (d, *J*=1.4 Hz, 2H_h_), 3.66–3.53 (m, 1H_e_), 3.26–3.06 (m, 2H_g_), 2.56 (t, *J*=7.4 Hz, 2H_a_), 2.48 (m, 1H_f_), 1.93 (m, 1H_f_), 1.74 (m, 4H), 1.60 (m, 2H_c_). ^13^C NMR (101 MHz, CDCl_3_) δ 171.46, 150.17, 133.65, 126.15, 124.88, 92.34, 56.40, 40.40, 38.68, 34.74, 34.16, 28.81, 24.71, 9.24. HRMS (ESI‐ve) m/z: 582.87521 ([M+H]^+^, C_18_H_17_O_2_^127^I_2_^32^S_2_ requires 582.87538).

**Procedure A**: Cu(MeCN)_4_PF_6_ (0.2 equiv.) and tris(benzyltriazolylmethyl)amine (TBTA) (0.2 equiv.) were dissolved in degassed CH_2_Cl_2_ (ca. 2 mL) and left to stir for 10 minutes under an atmosphere of N_2_. The respective azides (3 equiv.) and alkynes (1 equiv.) were subsequently added to a solution of the copper complex. The resultant mixtures were left to stir at room temperature and were monitored by TLC analysis until full conversion of the respective alkyne and the mono‐triazole intermediates to the bis‐triazole products was observed. The resultant mixtures were diluted with CH_2_Cl_2_ (10 mL) and the organic layer washed with aqueous 0.01 M NH_4_OH/ EDTA solution (10 mL). The resultant aqueous layer was back extracted with CH_2_Cl_2_ (2×10 mL), the combined organic phases were dried over MgSO_4_ and concentrated in vacuo to obtain the crude product mixture, and the product isolated by silica‐gel column chromatography.

**Synthesis of receptor 1.HB**: Synthesised according to Procedure A: **4 a** (70 mg, 0.212 mmol), azido‐methylferrocene (153 mg, 0.636 mmol), Cu(MeCN)_4_PF_6_ (16 mg, 0.042 mmol), TBTA (23 mg, 0.042 mmol). **1.HB** isolated as orange solid (72 mg, 0.178 mmol, 84 %). ^1^H NMR (500 MHz, CDCl_3_) δ 8.04 (s, 1H_i_), 7.70 (s, 2H_j_), 7.48 (d, *J*=1.5 Hz, 2H_h_), 5.32 (s, 4H_k_), 4.34–4.26 (m, 4H_l_), 4.26–4.22 (m, 4H_m_), 4.19 (s, 10H_n_), 3.69–3.52 (m, 1H_e_), 3.23–3.08 (m, 2H_g_), 2.57 (t, *J*=7.4 Hz, 2H_a_), 2.52–2.42 (m, 1H_f_), 1.98–1.88 (m, 1H_f_), 1.84–1.69 (m, 4H_b,d_), 1.63–1.50 (m, 2H_c_).^13^C NMR (126 MHz, CDCl_3_) δ 171.88, 151.68, 146.60, 132.76, 120.10, 119.67, 118.42, 80.60, 69.38, 69.15, 69.07, 56.42, 50.38, 40.36, 38.67, 34.71, 34.26, 29.82, 28.83, 24.78. HRMS (ESI+ve) m/z: 812.13451 ([M+H]^+^, C_40_H_40_O_2_N_6_^56^Fe_2_^32^S_2_ requires 812.13475).

**Synthesis of receptor 1.XB**: Synthesised according to Procedure A: **4 b** (70 mg, 0.120 mmol), azido‐methylferrocene (87 mg, 0.36 mmol), Cu(MeCN)_4_PF_6_ (9 mg, 0.024 mmol), TBTA (13 mg, 0.024 mmol). **1.XB** isolated as orange solid (88 mg, 0.083 mmol, 69 %).^1^H NMR (500 MHz, CDCl_3_) δ 8.44 (t, *J*=1.6 Hz, 1H_i_), 7.73 (d, *J*=1.5 Hz, 2H_h_), 5.41 (s, 4H_j_), 4.41 (t, *J*=1.9 Hz, 4H_k_), 4.20 (s, 10H_m_), 4.17 (t, *J*=1.9 Hz, 4H_l_), 3.68–3.52 (m, 1H_e_), 3.21–3.01 (m, 2H_g_), 2.60 (t, *J*=7.3 Hz, 2H_a_), 2.53–2.39 (m, 1H_f_), 1.97–1.85 (m, 1H_f_), 1.83–1.70 (m, 4H_b,d_), 1.62–1.46 (m, 2H_c_).^13^C NMR (126 MHz, CDCl_3_) δ 171.76, 151.06, 148.53, 132.12, 123.20, 120.71, 81.39, 76.49, 69.36, 69.07, 68.84, 56.49, 50.77, 40.37, 38.65, 34.69, 34.27, 29.82, 28.79, 24.80. HRMS (ESI+ve) m/z: 1063.92842 ([M+H]^+^, C_40_H_38_O_2_N_6_^56^Fe_2_^127^I_2_^32^S_2_ requires 1063.92804).

**Titration procedure**: Titrations were performed with **1.XB/HB_SAM_** confined to the Au disc electrode surface or 100 μM **1.XB/HB_dif_** under diffusive conditions in the chosen solvent system of ACN with varying water content, with 100 mM TBAClO_4_ as a supporting electrolyte. The ionic strength (and host concentration in diffusive experiments) was kept constant at 100 mM throughout by sequential additions of 100 mM TBAX (X= anion) up to a final anion concentration of 50 mM anion in all cases. The change in the receptors’ E_1/2_ was monitored by SWV (step potential: 2 mV, amplitude: 20 mV, frequency: 25 Hz).

## Conflict of interest

The authors declare no conflict of interest.

## Supporting information

As a service to our authors and readers, this journal provides supporting information supplied by the authors. Such materials are peer reviewed and may be re‐organized for online delivery, but are not copy‐edited or typeset. Technical support issues arising from supporting information (other than missing files) should be addressed to the authors.

Supplementary
