# Supplementary material for: Solvent Effects in Halogen and Hydrogen Bonding Mediated Electrochemical Anion Sensing in Aqueous Solution and at Interfaces
Source: Chemistry. 2021 May 19;27(39):10201–9. doi: 10.1002/chem.202101102 (PMC8360193; doi:10.1002/chem.202101102)
Supplement: Supplementary file 1 — Supplementary [file CHEM-27-10201-s001.pdf]

Chemistry–A European Journal

Supporting Information

Solvent Effects in Halogen and Hydrogen Bonding Mediated Electrochemical Anion Sensing in Aqueous Solution and at Interfaces

Sophie C. Patrick, Robert Hein, Andrew Docker, Paul D. Beer,* and Jason J. Davis*

__**Table of Contents:**__

S1 Materials and instrumentation 3

S1.1 General Information 3

S1.2 Electrochemical Measurements 3

S1.3 Electrode Cleaning Procedure 4

S1.4 SAM Formation 4

S1.5 Surface Characterisation 4

S1.6 Capacitance Measurements 5

S1.7 Data Analysis and Fitting of Binding Isotherms 5

S2 Synthesis and characterisation of 1.XB/HB 6

S3 Diffusive electrochemical anion sensing (1.XB/HB$_{dif}$) 17

S3.1 Electrochemical Characterisation 17

S3.2 Anion Sensing Studies with **1.XB/HB$_{dif}$** and Comparisons with **2.XB/HB$_{dif}$** 20

S4 Solvent Dependency of 1.XB/HB$_{SAM/dif}$ 26

S5 Interfacial electrochemical anion sensing (1.XB/HB$_{dif}$) 31

S5.1 Surface Characterisation 31

S5.2 Electrochemical Surface Characterisation 33

S5.3 Anion Sensing Studies with **1.XB/HB$_{SAM}$** 34

S6 Maximum voltammetric shifts, BEF (BEF$_{maxshift}$ and BEF$_{Fit}$) and SEF data 36

S6.1 Maximum Cathodic Shifts for **1.XB/HB$_{dif/SAM}$** 36

S6.2 Binding Enhancement Factors (BEFs) from ΔE_{max} and 1:1 Host-guest Nernst Model for **1.XB/HB$_{dif}$** 37

S6.3 Binding Enhancement Factors (BEFs) from ΔE_{max} and 1:1 Host-guest Nernst Model for **1.XB/HB$_{SAM}$** 41

S6.4 Surface Enhancement Factors (SEFs) for **1.XB/HB$_{dif/SAM}$** 44

S7 References 47

S1: Materials and instrumentation

S1.1 General Information:

All experiments were completed at room temperature in the presence of oxygen, unless stated otherwise. All chemicals and solvents were obtained from commercial suppliers and used as received unless noted otherwise. Supporting electrolyte ($TBAClO_4$ from Sigma Aldrich) was of electrochemical grade. All hygroscopic TBA salts were stored in vacuum dessicators at room temperature. Anhydrous solvents were degassed with N_2 and dried on a Mbraun MPSP-800 column. Ultrapure water was drawn from a Milli-Q system (18.2 MΩcm). Electrochemical measurements were performed with an Autolab Potentiostat (Metrohm). All measurements were carried out in a 3-electrode cell equipped with a Au disc (or glassy carbon disc electrode for studies under diffusive conditions, BASi 3 mm diameter) working electrode (BASi or IJ Cambria; 1.6 or 2 mm diameter), a platinum wire counter electrode and either a Ag|AgCl (3 M KCl) or non-aqueous Ag|$AgNO_3$ reference electrode, depending on the solvent used. Unless stated otherwise, all potentials are wrt. non-aqueous Ag|$AgNO_3$ reference electrode. Mass spectrometry was performed on a Bruker micrOTOF. NMR spectra were recorded on Bruker NMR spectrometers (AVIII HD 500 or AVIII HD 400)).

S1.2 Electrochemical Measurements

Electrochemical measurements were performed with an Autolab Potentiostat (Metrohm). All measurements were carried out in a 3-electrode cell equipped with a Au disc working electrode for interfacial studies (BASi or IJ Cambria; 1.6 or 2 mm diameter), or glassy carbon disc electrode for studies under diffusive conditions (BASi 3 mm diameter), a platinum wire counter electrode and either a Ag|AgCl (3 M KCl) or non-aqueous Ag|$AgNO_3$ reference electrode, depending on the solvent used. Unless stated otherwise, all potentials are wrt. non-aqueous Ag|$AgNO_3$ reference electrode. The half-wave potentials were determined as the peak potential by SWV.

S1.3 Electrode Cleaning Procedure

Au disc electrodes and glassy carbon electrode were polished mechanically with a 0.05 µm alumina slurry for 2 min, followed by sonication in 1:1 EtOH : H_2O for several minutes. The Au disc electrodes were then chemically polished by submersion in fresh piranha acid (3:1 conc. H_2SO_4: 30% H_2O_2), and subsequently electrochemically polished in 0.5 M KOH from -0.7 V to -1.7 V, then in 0.5 M H_2SO_4 from -0.15 V to 1.35 V for at least 1 hour each (at a scan rate of 100 mV s^{-1}, potentials wrt to aqueous Ag|AgCl (3 M KCl) reference electrode).

S1.4 SAM Formation

Immediately following electrochemical polishing, the Au disc electrodes were rinsed thoroughly with copious amounts of water and ethanol, dried under a stream of N_2 and submerged in the receptor solution. Formation of SAMs of the receptors was carried out by immersion of the Au disc electrodes in 1 mM **1.XB/HB** solutions in DCM overnight in the dark, followed by thorough washing with DCM, yielding **1.XB/HB**$_{\mathbf{SAM}}$.

S1.5 Surface characterisation:

SAMs for ellipsometry or IR analyses were formed in the same manner as on gold disc electrodes (1 mM receptor in DCM) but on larger gold on silicon substrates (prepared in-house), which were cleaned by immersion in fresh piranha solution (3:1 conc. H_2SO_4: 30% H_2O_2) and copious rinsing with EtOH and H_2O.

FT-ATR-IR spectra were measured on an IRTracer-100 (Shimadzu). Water contact angles measurements were recorded with a FTA1000B goniometer (First Ten Ångstroms). All ellipsometry measurements were performed on a Beaglehole Instruments Picometer equipped with a 2 mW Helium-Neon laser (632.8 nm) and average film thicknesses and standard deviations were calculated from five different measurements on the same substrate.

S1.6 Capacitance Measurements:

The film capacitance, C was obtained by electrochemical impedance spectroscopy (EIS) in 100 mM $NaPF_6$ (aq) vs. Ag|AgCl (3 M KCl) reference electrode, which was recorded between 40 kHz and 0 Hz for 40 frequencies which were logarithmically stepped, using a 10 mV amplitude and 0 V as the DC potential. C was then used in conjunction with measured film thicknesses, d (as determined via ellipsometry), the surface area of the Au working electrode, A and the permittivity of free space, $\boldsymbol{\varepsilon_0}$ to calculate the dielectric constant of the film, $\boldsymbol{\varepsilon_r}$ via the relationship described by eqn S1.[1]

$$C = \frac{(\varepsilon_0 \times \varepsilon_r \times A)}{d} \qquad \text{(Eqn S1)}$$

S1.7 Data Analysis and Fitting of Binding Isotherms

All data analysis and fitting was carried out with OriginPro 2017. Analysis of the sensor responses was carried out via eqns. 1-2. Fitting of the binding isotherms according two eqn. 2 was carried out without any restriction of the parameters unless stated otherwise. In some cases, nonsensical values were obtained from fits with eqn. 2 (e.g. negative values for K_{red}, Tables S7-9) which is chemically not possible, and is purely the result of the fitting (affording the best mathematical fit to eqn. 2). Fits can be restricted to K_{Red}=0 which generally affords similar fits.

$$\Delta E = -\frac{RT}{nF}\ln\left(\frac{K_{Ox}}{K_{Red}}\right) \qquad \text{(Eqn 1)}$$

$$\Delta E = -\frac{RT}{nF}\ln\left(\frac{1 + K_{Ox}[A^-]}{1 + K_{Red}[A^-]}\right) \qquad \text{(Eqn 2)}$$

Further details about electrochemical measurements, electrode cleaning protocols, SAM formation and titration procedures are detailed in the experimental section of the main manuscript.

S2. Synthesis and Characterisation of 1.XB/HB

Procedure A:

$Cu(MeCN)_4PF_6$ (0.2 equiv.) and tris(benzyltriazolylmethyl)amine (TBTA) (0.2 equiv.) were dissolved in degassed CH_2Cl_2 (ca. 2 mL) and left to stir for 10 minutes under an atmosphere of N_2. The respective azides (3 equiv.) and alkynes (1 equiv.) were subsequently added to a solution of the copper complex. The resultant mixtures were left to stir at room temperature and were monitored by TLC analysis until full conversion of the respective alkyne and the mono-triazole intermediates to the bis-triazole products was observed. The resultant mixtures were diluted with CH_2Cl_2 (10 mL) and the organic layer washed with aqueous 0.01 M NH_4OH/ EDTA (ethylenediaminetetraacetic acid) solution (10 mL). The resultant aqueous layer was back extracted with CH_2Cl_2 (2 x 10 mL), the combined organic phases were dried over $MgSO_4$ and concentrated in vacuo to obtain the crude product mixture, and the product isolated by silica-gel column chromatography.

Synthesis of 3b:

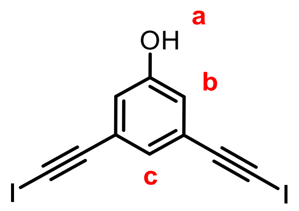

3b

3,5-diethynyl phenol **3a** (500 mg, 3.52 mmol) (synthesised according to literature[2]), N-Iodomorpholine hydriodide (3.61 g, 10.6 mmol) and CuI (67 mg , 0.352 mmol) were dissolved in anhydrous tetrahydrofuran (THF) (22 ml) and left to stir excluded from the presence of light for 4 h at room temperature. Afterwards the reaction mixture was diluted with CH_2Cl_2 (100 ml) and filtered through a CH_2Cl_2 saturated alumina pad. The organic phase subsequently washed with 0.01 M NH_4OH/EDTA solution, dried over $MgSO_4$ filtered and solvent removed in vacuo. The crude material was subjected to silica-gel column chromatography and isolated as a white solid (792 mg, 2.01 mmol, 57%). **^{1}H NMR** (400 MHz, $CDCl_3$) δ 7.08 (t, *J* = 1.4 Hz, $1H_c$), 6.86 (d, *J* = 1.4 Hz, $2H_b$), 5.22 (s, $1H_a$).

^{13}C NMR (101 MHz, $CDCl_3$) δ 155.19, 129.13, 124.84, 119.92, 92.91, 7.95.

HRMS (ESI-ve) m/z: 392.82748 ($[M-H]^-$, $C_{10}H_3O^{127}I_2$ requires 392.82787).

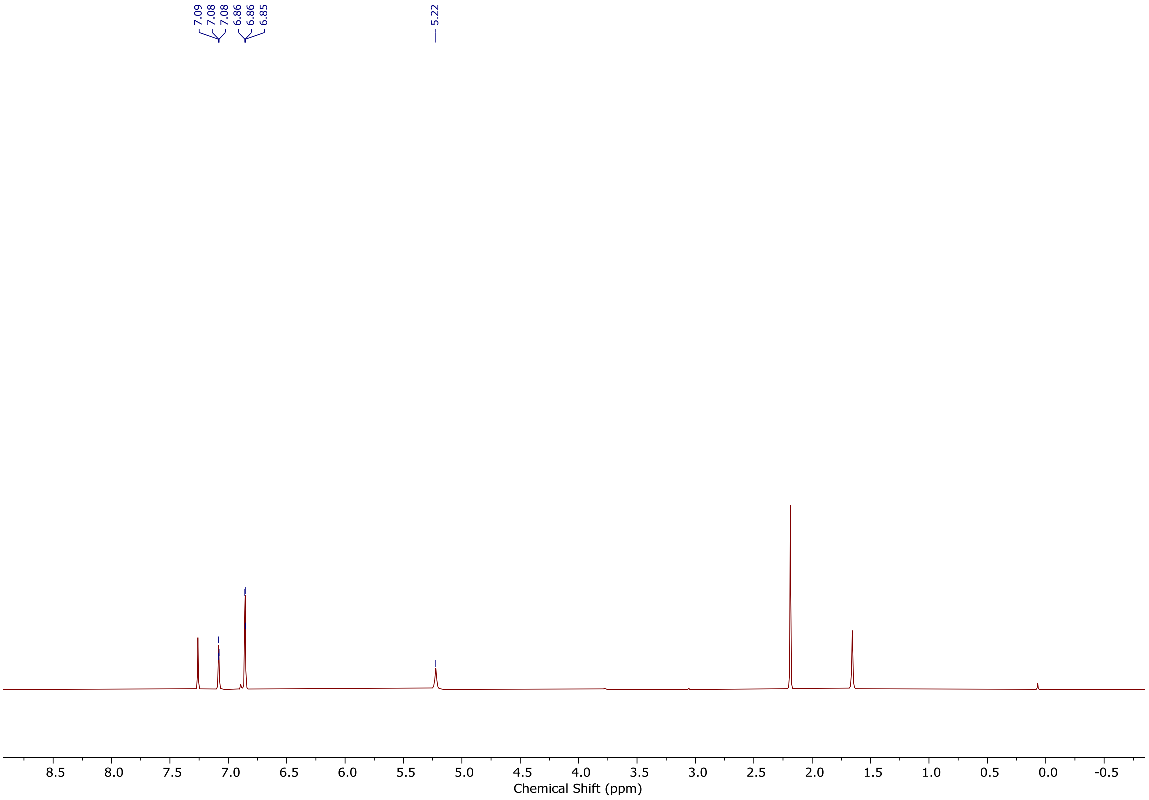

Figure S1. ^{1}H NMR spectrum of **3b**.

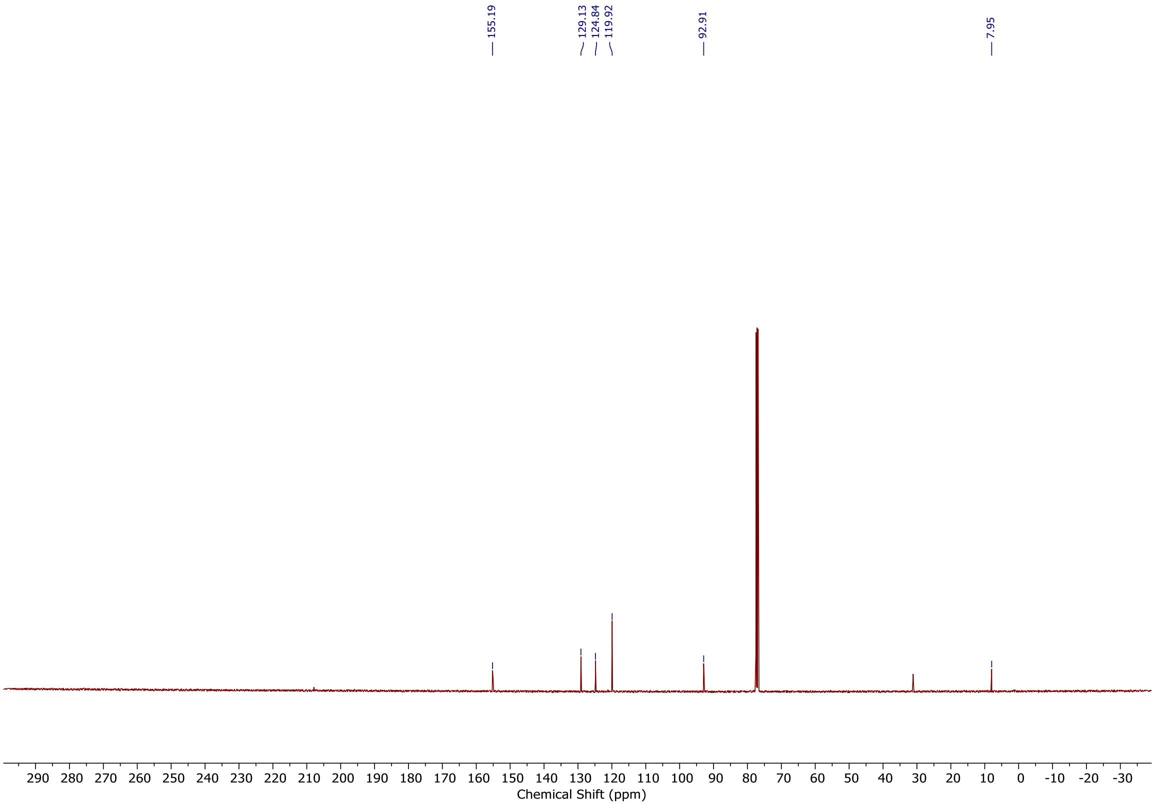

Figure S2. ^{13}C NMR spectrum of **3b**.

Synthesis of 4a:

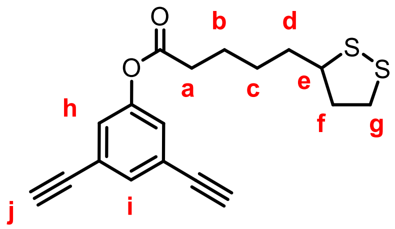

4a

3,5-diethynyl phenol **3a** (100 mg, 0.704 mmol), lipoic acid (174 mg, 0.845 mmol), dicyclohexylcarbodiimide (192 mg, 0.930 mmol) and ca. 5 mg of 4-dimethylaminopyridine (DMAP) were dissolved in anhydrous CH_2Cl_2 (20 ml) and left to stir at room temperature. After 2 hours the mixture was diluted with CH_2Cl_2 (10 ml) filtered and the resultant filtrate was concentrated in vacuo. The crude residue was subjected to silica-gel column chromatography and isolated as a yellow solid (233 mg, 0.704 mmol, quantitative).

^{1}H NMR (500 MHz, $CDCl_3$) δ 7.46 (s, $1H_i$), 7.23 – 7.18 (m, $2H_h$), 3.65 – 3.53 (m, $1H_e$), 3.25 – 3.03 (m, $4H_{g,j}$), 2.57 (t, J = 7.5 Hz, $2H_a$), 2.52 – 2.42 (m, $1H_f$), 2.00 – 1.85 (m, $1H_f$), 1.84 – 1.66 (m, $4H_{b,d}$), 1.64 – 1.50 (m, $2H_c$).

^{13}C NMR (126 MHz, $CDCl_3$) δ 171.49, 150.34, 133.22, 125.94, 123.81, 81.78, 78.92, 56.37, 40.36, 38.65, 34.70, 34.13, 28.78, 24.67.

HRMS (ESI+ve) m/z: 331.08228 ($[M+H]^+$, $C_{18}H_{19}O_2{}^{32}S_2$ requires 331.08210).

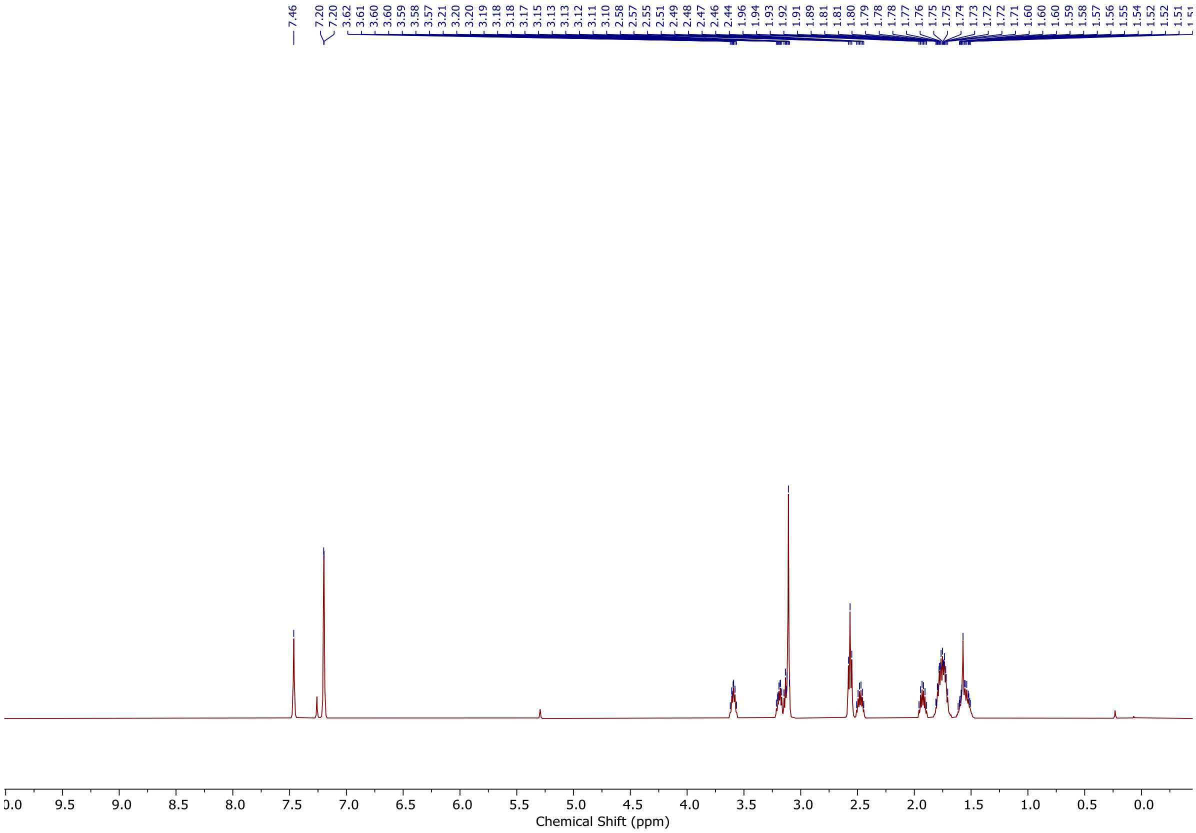

Figure S3. ^{1}H NMR spectrum of **4a**.

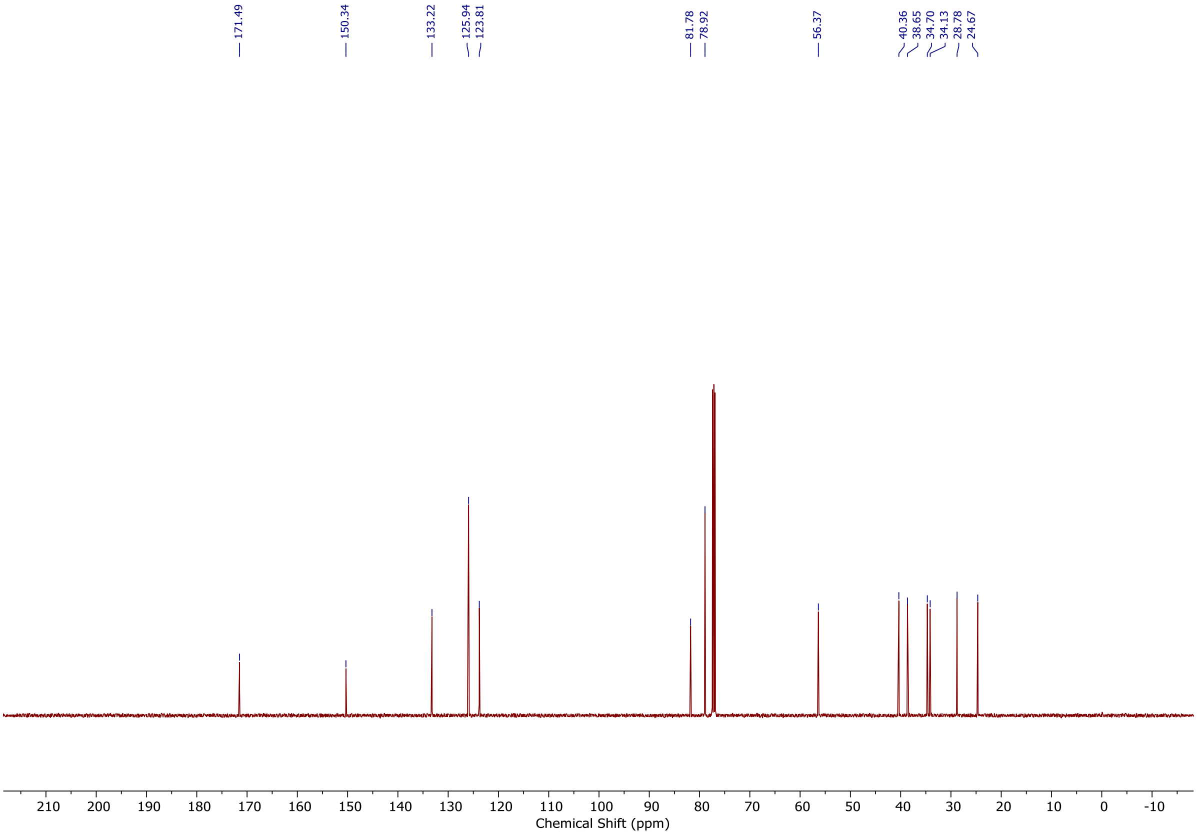

Figure S4. ^{13}C NMR spectrum of **4a**.

Synthesis of 4b:

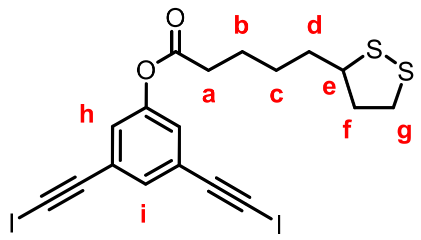

4b

3b (200 mg, 0.508 mmol), lipoic acid (126 mg, 0.609 mmol), dicyclohexylcarbodiimide (115 mg, 0.558 mmol) and ca. 5 mg of DMAP were dissolved in anhydrous CH_2Cl_2 (20 ml) and left to stir at room temperature. After 2 hours the mixture was diluted with CH_2Cl_2 (10 ml) filtered and the resultant filtrate was concentrated in vacuo. The crude residue was subjected to silica-gel column chromatography and isolated as a yellow solid (266 mg, 0.457 mmol, 90%).

^{1}H NMR (400 MHz, $CDCl_3$) δ 7.35 (t, *J* = 1.4 Hz, $1H_i$), 7.12 (d, *J* = 1.4 Hz, $2H_h$), 3.66 – 3.53 (m, $1H_e$), 3.26 – 3.06 (m, $2H_g$), 2.56 (t, *J* = 7.4 Hz, $2H_a$), 2.48 (m, $1H_f$), 1.93 (m, $1H_f$), 1.74 (m, 4H), 1.60 (m, $2H_c$).

^{13}C NMR (101 MHz, $CDCl_3$) δ 171.46, 150.17, 133.65, 126.15, 124.88, 92.34, 56.40, 40.40, 38.68, 34.74, 34.16, 28.81, 24.71, 9.24.

HRMS (ESI-ve) m/z: 582.87521 ($[M+H]^+$, $C_{18}H_{17}O_2{}^{127}I_2{}^{32}S_2$ requires 582.87538).

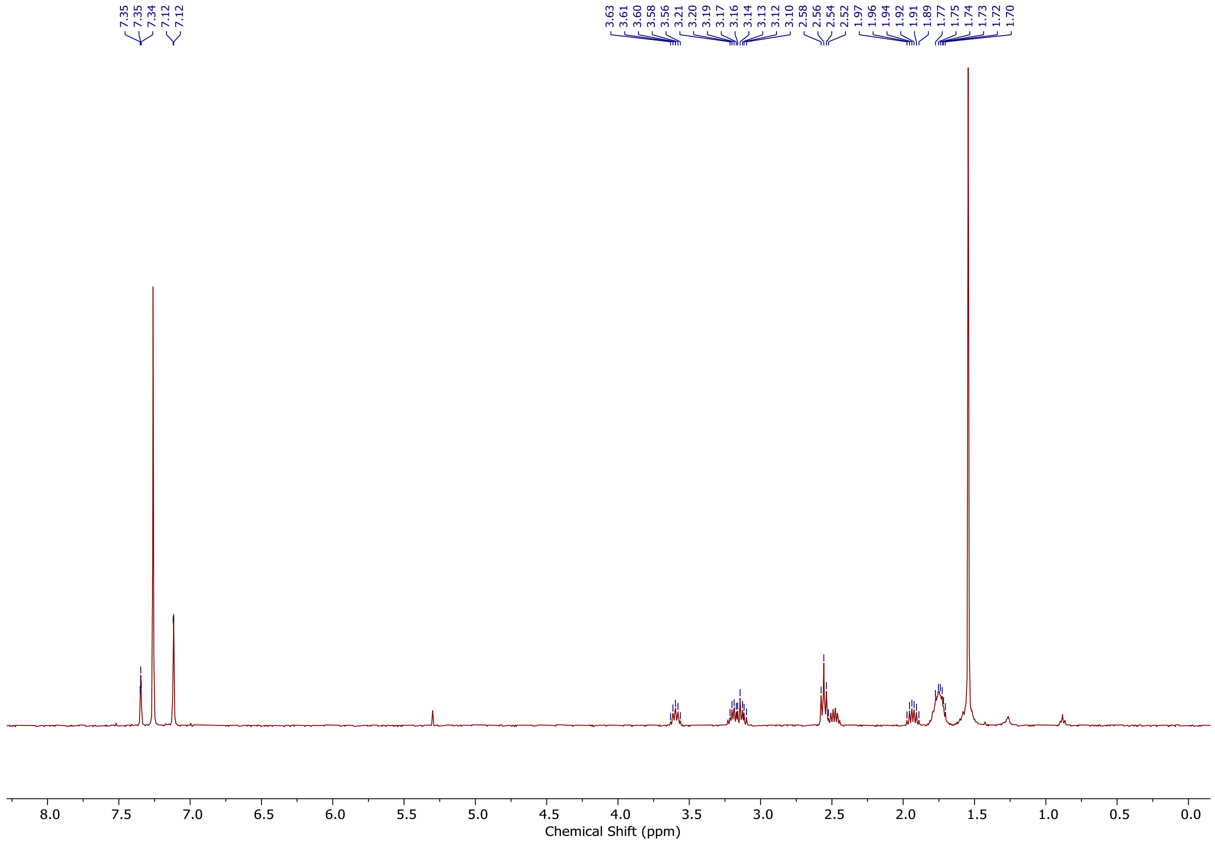

Figure S5. ^{1}H NMR spectrum of **4b**.

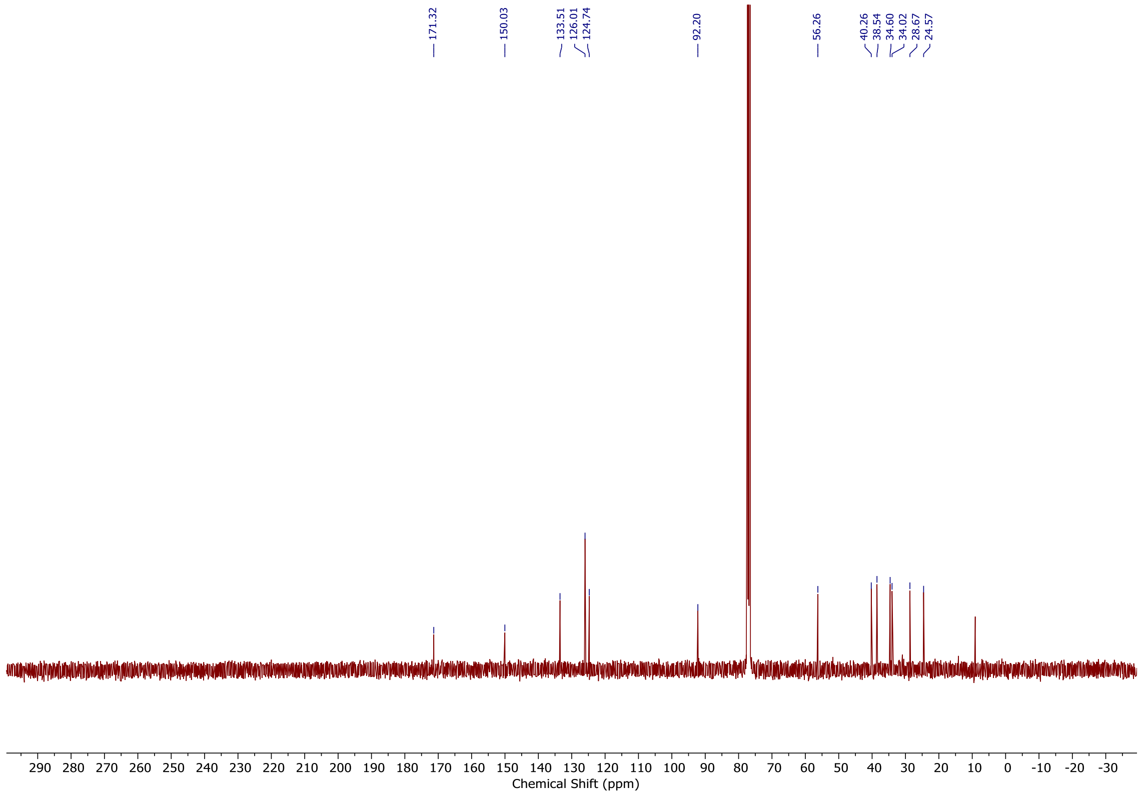

Figure S6. ^{13}C NMR spectrum of **4b**.

Synthesis of 1.HB:

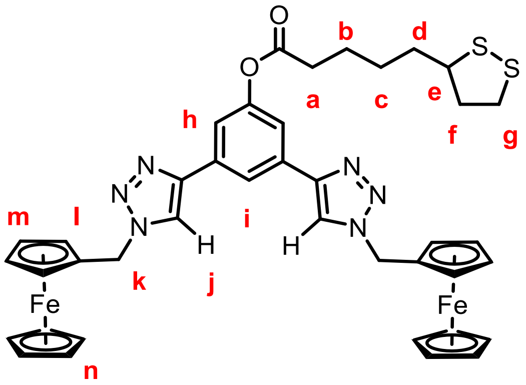

1.HB

Synthesised according to Procedure A: **4a** (70 mg, 0.212 mmol), azido-methylferrocene (153 mg, 0.636 mmol), $Cu(MeCN)_4PF_6$ (16 mg, 0.042 mmol), TBTA (23 mg, 0.042 mmol). **1.HB** isolated as orange solid (72 mg, 0.178 mmol, 84%).

^{1}H NMR (500 MHz, $CDCl_3$) δ 8.04 (s, $1H_i$), 7.70 (s, $2H_j$), 7.48 (d, J = 1.5 Hz, $2H_h$), 5.32 (s, $4H_k$), 4.34 – 4.26 (m, $4H_l$), 4.26 – 4.22 (m, $4H_m$), 4.19 (s, $10H_n$), 3.69 – 3.52 (m, $1H_e$), 3.23 – 3.08 (m, $2H_g$), 2.57 (t, J = 7.4 Hz, $2H_a$), 2.52 – 2.42 (m, $1H_f$), 1.98 – 1.88 (m, $1H_f$), 1.84 – 1.69 (m, $4H_{b,d}$), 1.63 – 1.50 (m, $2H_c$).

^{13}C NMR (126 MHz, $CDCl_3$) δ 171.88, 151.68, 146.60, 132.76, 120.10, 119.67, 118.42, 80.60, 69.38, 69.15, 69.07, 56.42, 50.38, 40.36, 38.67, 34.71, 34.26, 29.82, 28.83, 24.78.

HRMS (ESI+ve) m/z: 812.13451 ($[M+H]^+$, $C_{40}H_{40}O_2N_6\,^{56}Fe_2\,^{32}S_2$ requires 812.13475).

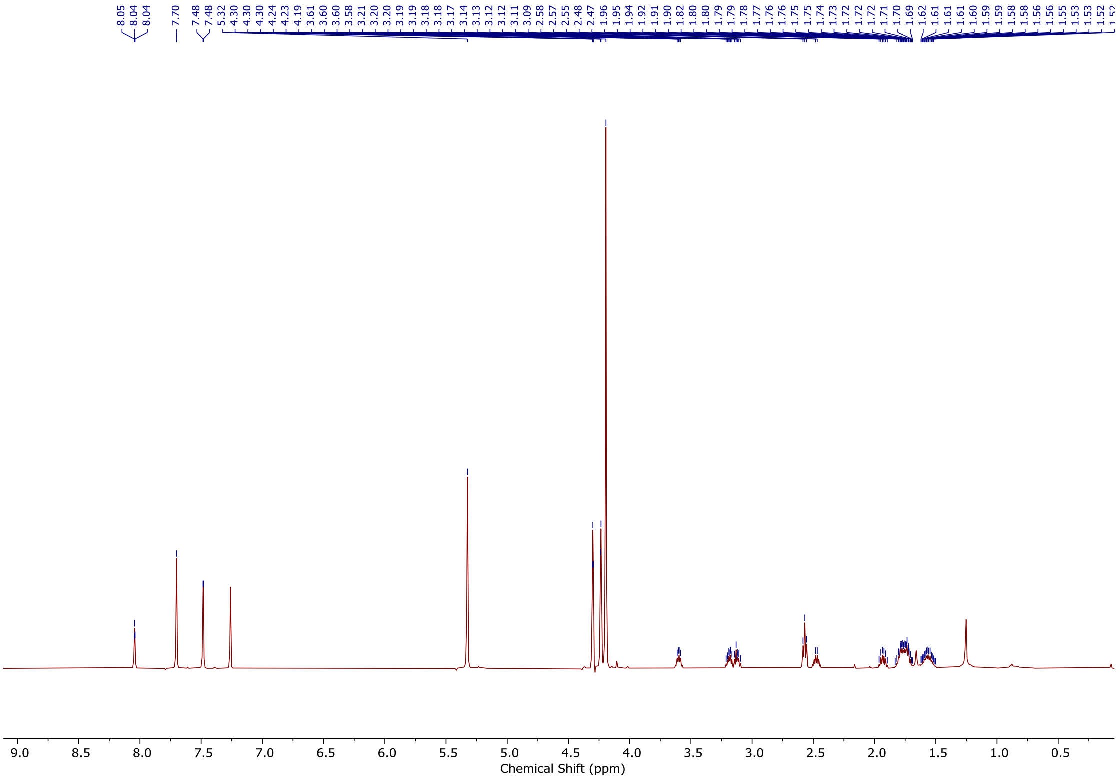

Figure S7. ^{1}H NMR spectrum of **1.HB**.

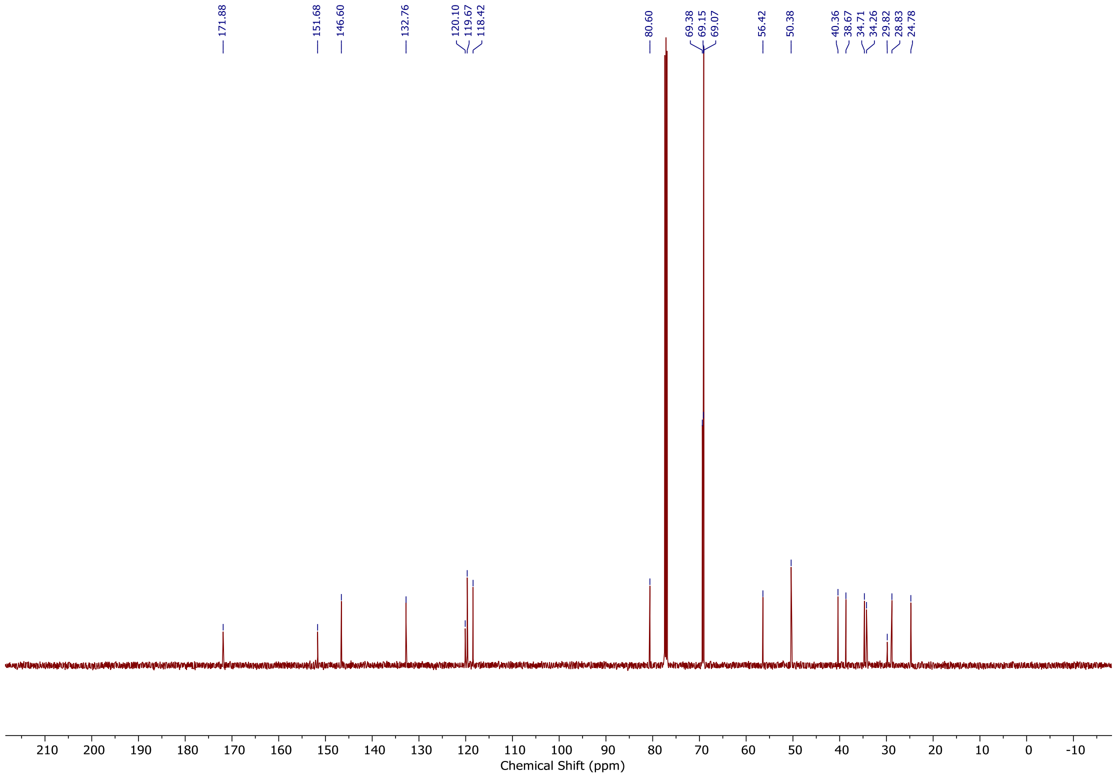

Figure S8. ^{13}C NMR spectrum of **1.HB**.

Synthesis of 1.XB:

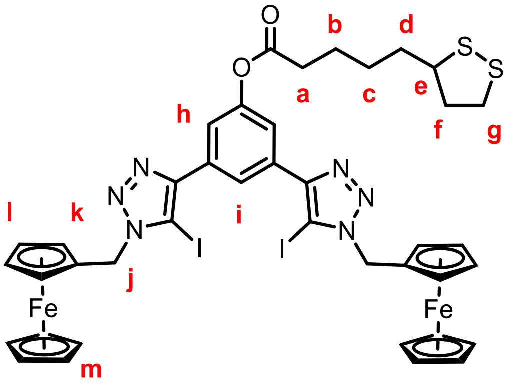

1.XB

Synthesised according to Procedure A: **4b** (70 mg, 0.120 mmol), azido-methylferrocene (87 mg, 0.36 mmol), $Cu(MeCN)_4PF_6$ (9 mg, 0.024 mmol), TBTA (13 mg, 0.024 mmol). **1.XB** isolated as orange solid (88 mg, 0.083 mmol, 69%).

^{1}H NMR (500 MHz, $CDCl_3$) δ 8.44 (t, *J* = 1.6 Hz, 1H_i), 7.73 (d, *J* = 1.5 Hz, 2H_h), 5.41 (s, 4H_j), 4.41 (t, *J* = 1.9 Hz, 4H_k), 4.20 (s, 10H_m), 4.17 (t, *J* = 1.9 Hz, 4H_l), 3.68 – 3.52 (m, 1H_e), 3.21 – 3.01 (m, 2H_g), 2.60 (t, *J* = 7.3 Hz, 2H_a), 2.53 – 2.39 (m, 1H_f), 1.97 – 1.85 (m, 1H_f), 1.83 – 1.70 (m, 4$H_{b,d}$), 1.62 – 1.46 (m, 2H_c).

^{13}C NMR (126 MHz, $CDCl_3$) δ 171.76, 151.06, 148.53, 132.12, 123.20, 120.71, 81.39, 76.49, 69.36, 69.07, 68.84, 56.49, 50.77, 40.37, 38.65, 34.69, 34.27, 29.82, 28.79, 24.80.

HRMS (ESI+ve) m/z: 1063.92842 ($[M+H]^+$, $C_{40}H_{38}O_2N_6\,^{56}Fe_2\,^{127}I_2\,^{32}S_2$ requires 1063.92804).

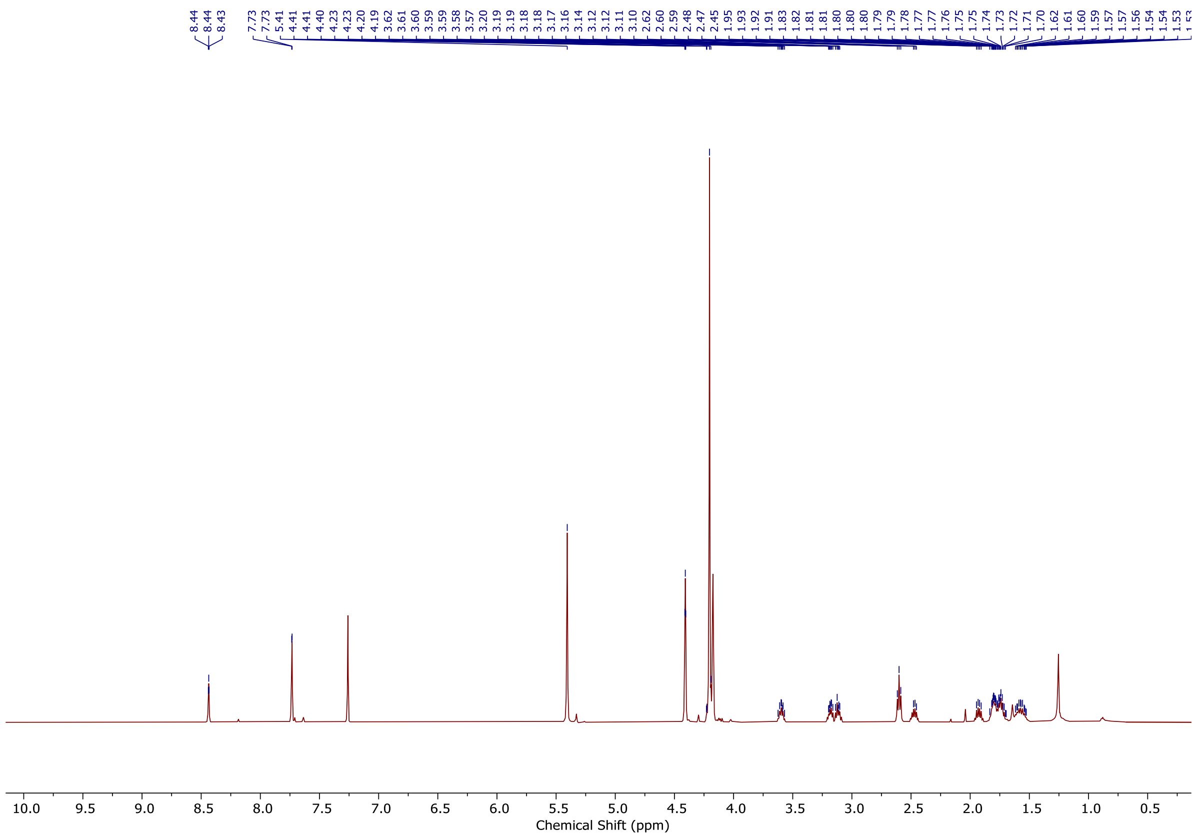

Figure S9. ^{1}H NMR spectrum of **1.XB**.

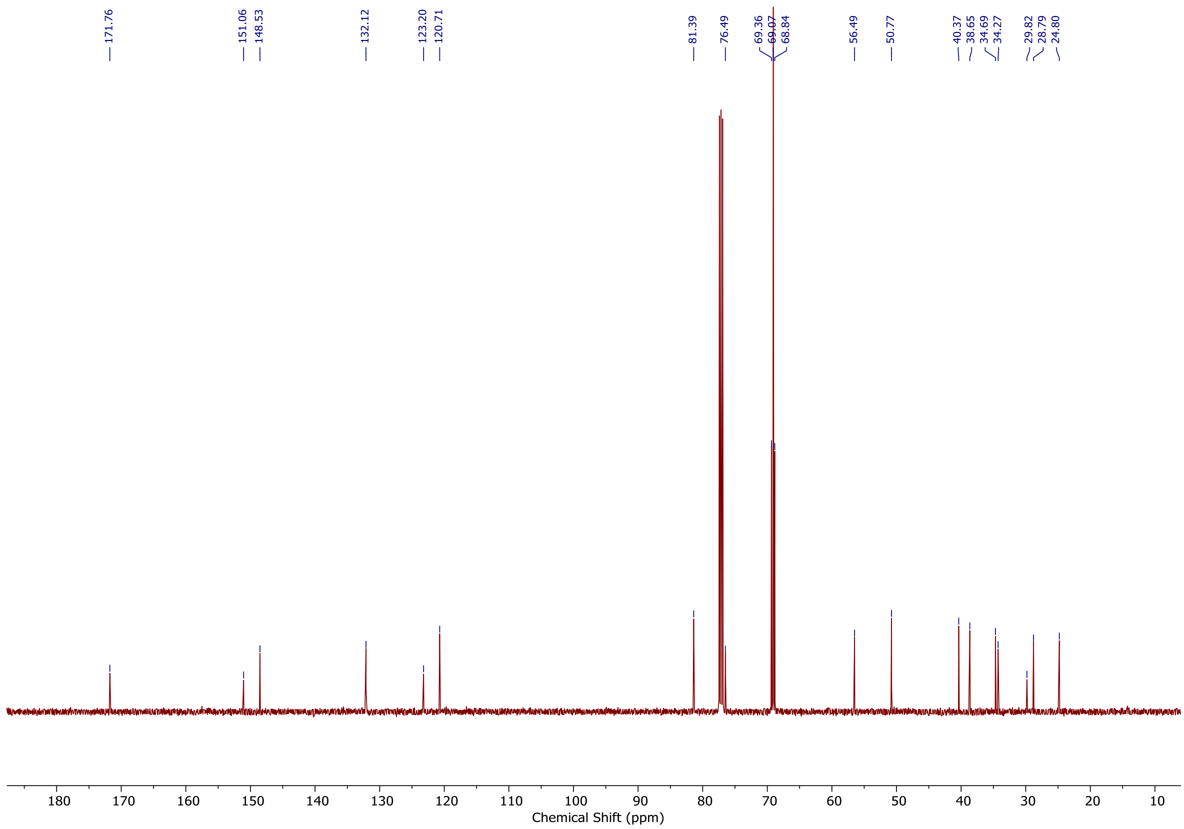

Figure S10. ^{13}C NMR spectrum of **1.XB**.

S3: Diffusive Electrochemical Anion Sensing

S3.1 Electrochemical characterisation:

A peak separation of 62 ± 1 mV and 57 ± 1 mV for of **1.XB/HB$_{dif}$**, respectively, and a close to unity value of the ratio of anodic and cathodic peak currents ascertained quasi-reversibility of the Fc|Fc^{+} couple. However, the linear dependence of the peak currents on the scan rate suggests some degree of physiorption of the receptors onto the glassy carbon working electrode surface (Figures S13, S14, S15).

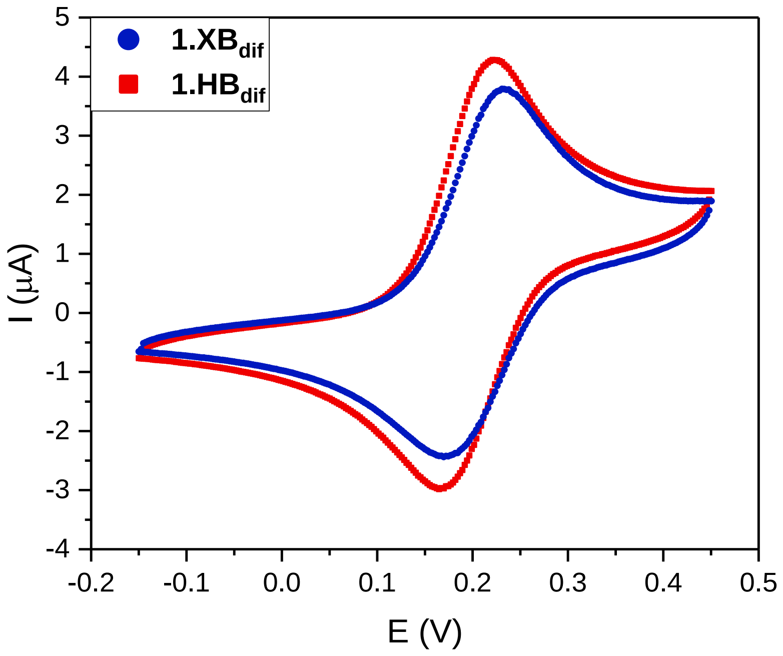

Figure S11. Cyclic voltammograms of 0.1 mM **1.XB/HB$_{dif}$** in ACN, 100 mM $TBAClO_4$, at a scan rate of 100 mV s^{-1}. Potentials wrt. Ag|$AgNO_3$.

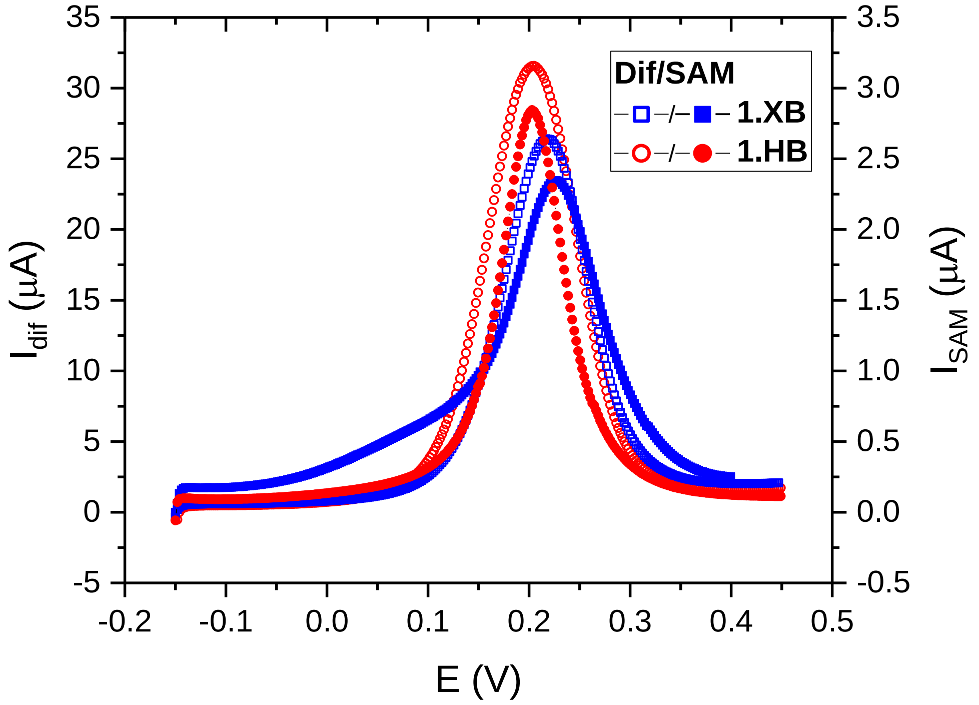

Figure S12. Square wave voltammograms of **1.XB/HB$_{dif}$** (0.1 mM) and **1.XB/HB$_{SAM}$** in ACN, 100 mM $TBAClO_4$, at a scan rate of 0.1 V s^{-1}. Potentials wrt. Ag|$AgNO_3$.

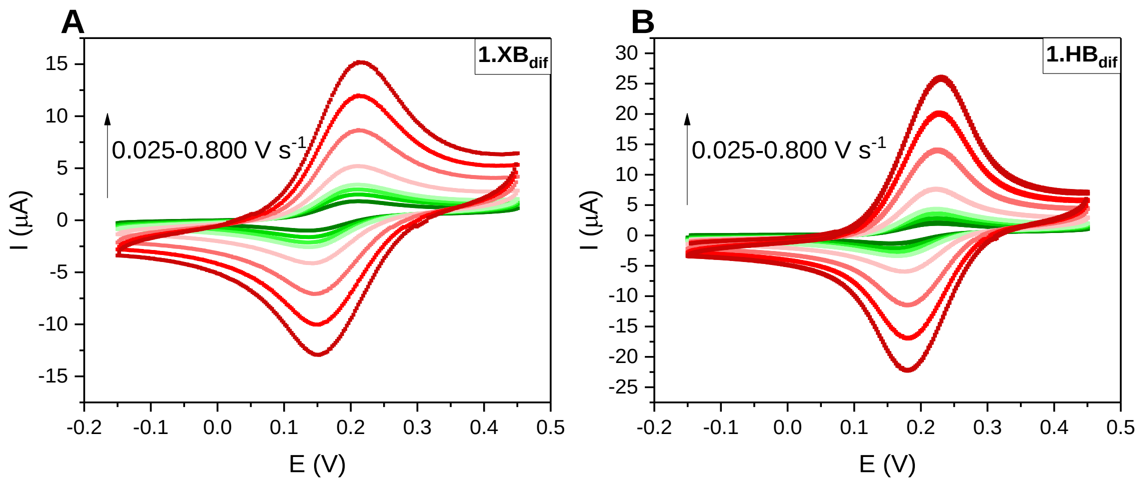

Figure S13. Cyclic voltammograms at varying scan rates from 0.025 to 0.800 V s^{-1} of 0.1 mM A) **1.HB$_{dif}$** and B) **1.XB$_{dif}$**. Potentials wrt. Ag|$AgNO_3$.

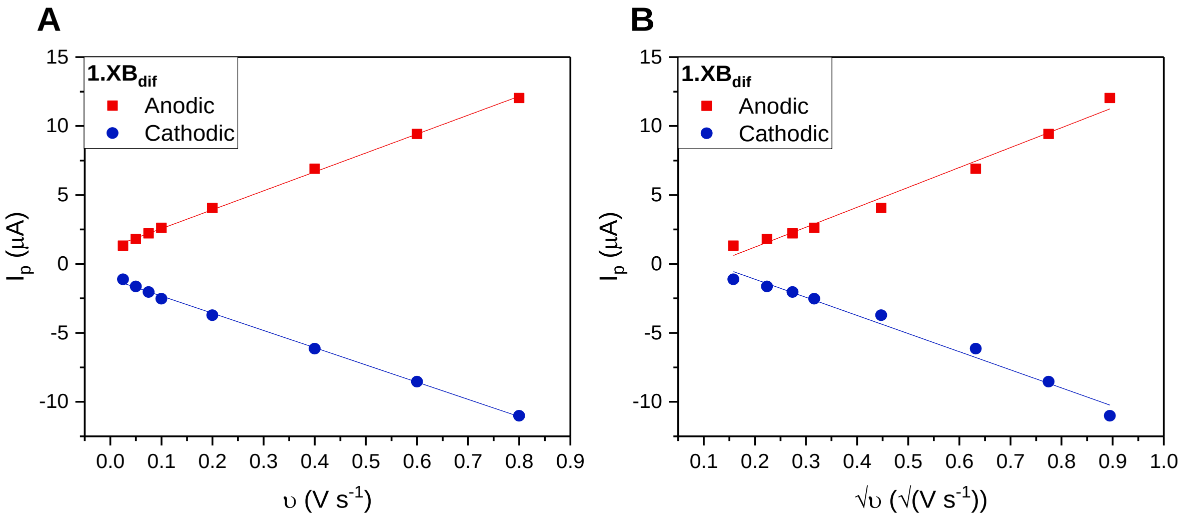

Figure S14. Peak current *vs.* A) scan rate B) the square-root of the scan rate with linear fits of 0.1 mM **$1.XB_{dif}$** in ACN (100 mM $TBAClO_4$).

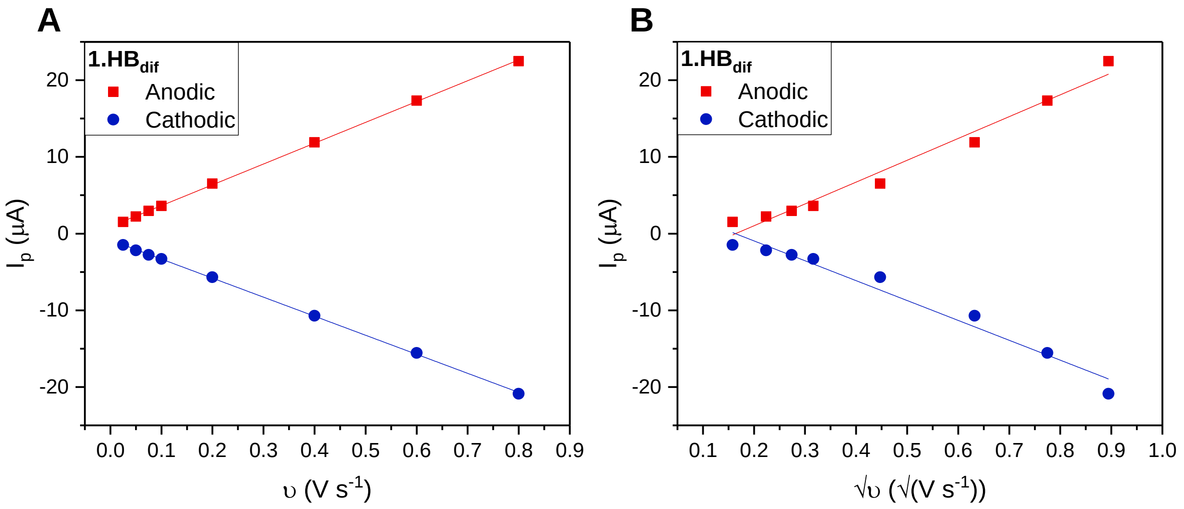

Figure S15. Peak current *vs.* A) scan rate B) the square-root of the scan rate with linear fits of 0.1 mM **$1.HB_{dif}$** in ACN (100 mM $TBAClO_4$).

S3.2 Anion Sensing Studies with 1.XB/HB$_{dif}$ and Comparisons with 2.XB/HB$_{dif}$:

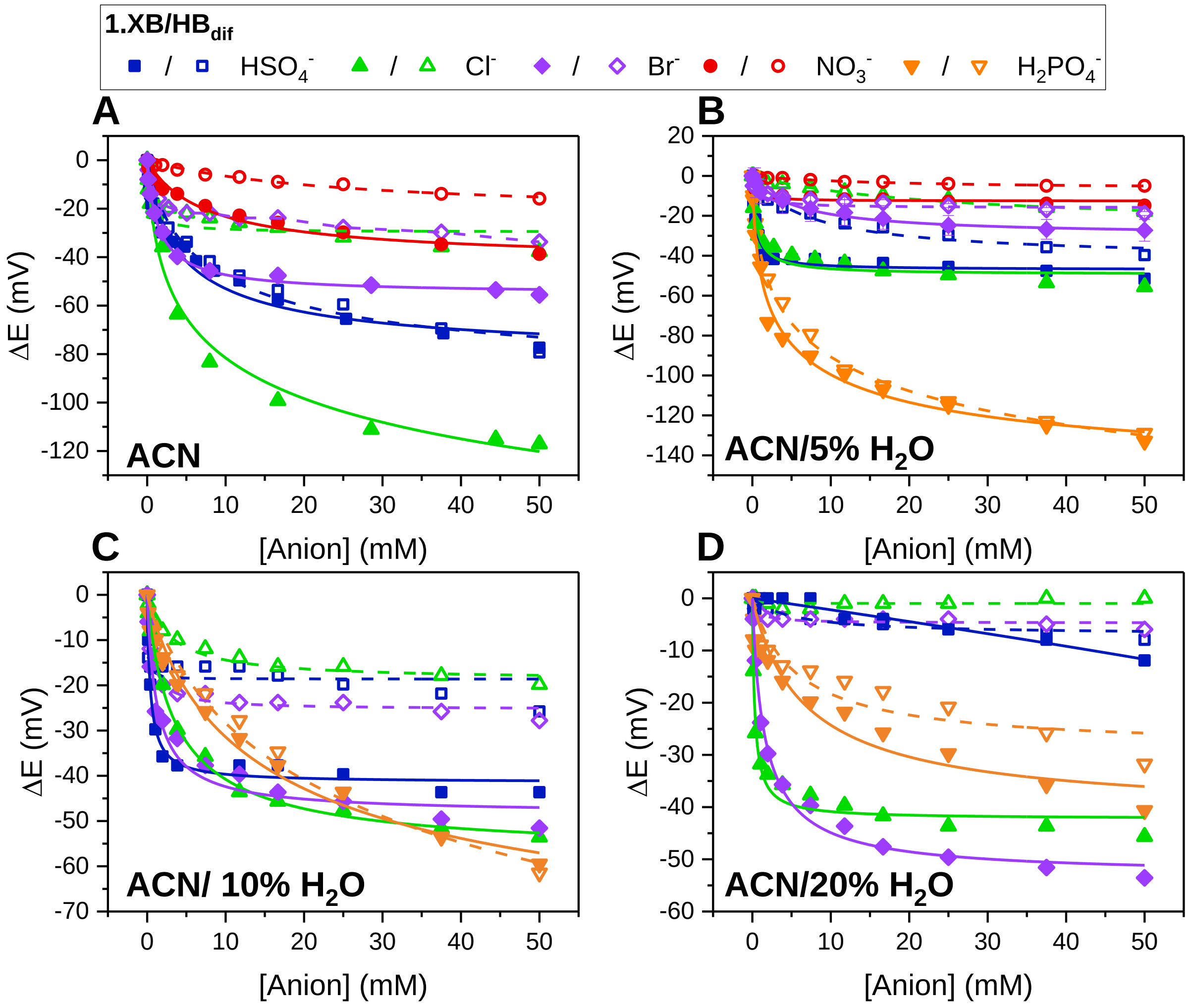

Figure S16. Cathodic voltammetric shifts of **1.XB/HB$_{dif}$** in A) ACN B) ACN/ 5% H_2O C) ACN/10% H_2O D) and ACN/20% H_2O upon titration with various anions. [**1.XB/HB**] = 0.1 mM with 100 mM $TBAClO_4$ supporting electrolyte. The overall ionic strength was kept constant at 100 mM throughout. Solid lines represent fits to a 1:1 host-guest Nernst model (eqn. 2). Note the different y-axis scaling for all graphs. Anions for which no isotherms are shown were predicted to induce negligible perturbations (i.e. NO_3^- in C and D) or caused significant transducer degradation (i.e. $H_2PO_4^-$ in ACN). Errors shown in (B) for Br^- represent one standard deviation of two independent measurements.

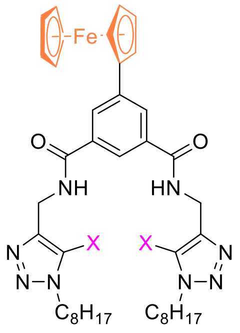

X= I or H

2.XB/HB$_{dif}$

Figure S17. Structure of **2.XB/HB$_{dif}$.**[3]

The general sensing properties of both **1.XB/HB$_{dif}$** compare favourably with recently reported Fc-isophthalamide-(iodo)triazole receptors **2.XB/HB$_{dif}$** (Figures S18 and S19).[3] For example, as shown in Figure S18A, **1.XB/HB$_{dif}$** displays slightly larger responses towards HSO_4^- in ACN. In this solvent **1.XB$_{dif}$** also significantly outperforms **2.XB$_{dif}$** in the sensing of Cl^- (Figure S18C), while **1.HB$_{dif}$** clearly outperforms **2.HB$_{dif}$** towards $H_2PO_4^-$ (Figure S19C). Particularly notable is also that **1.XB$_{dif}$** displays cathodic responses towards Cl^- and HSO_4^- in ACN/5% H_2O that are very similar to that of **2.XB$_{dif}$** in the much less competitive ACN/1% H_2O. These observations can most likely be attributed to a better spatial/electronic coupling of the Fc-groups and the anion binding site in **1.XB/HB$_{dif}$** as well as the presence of two redox transducers, enabling better redox-switching (**1.XB/HB$_{dif}^{2+}$** vs. **2.XB/HB$_{dif}^{+}$**).

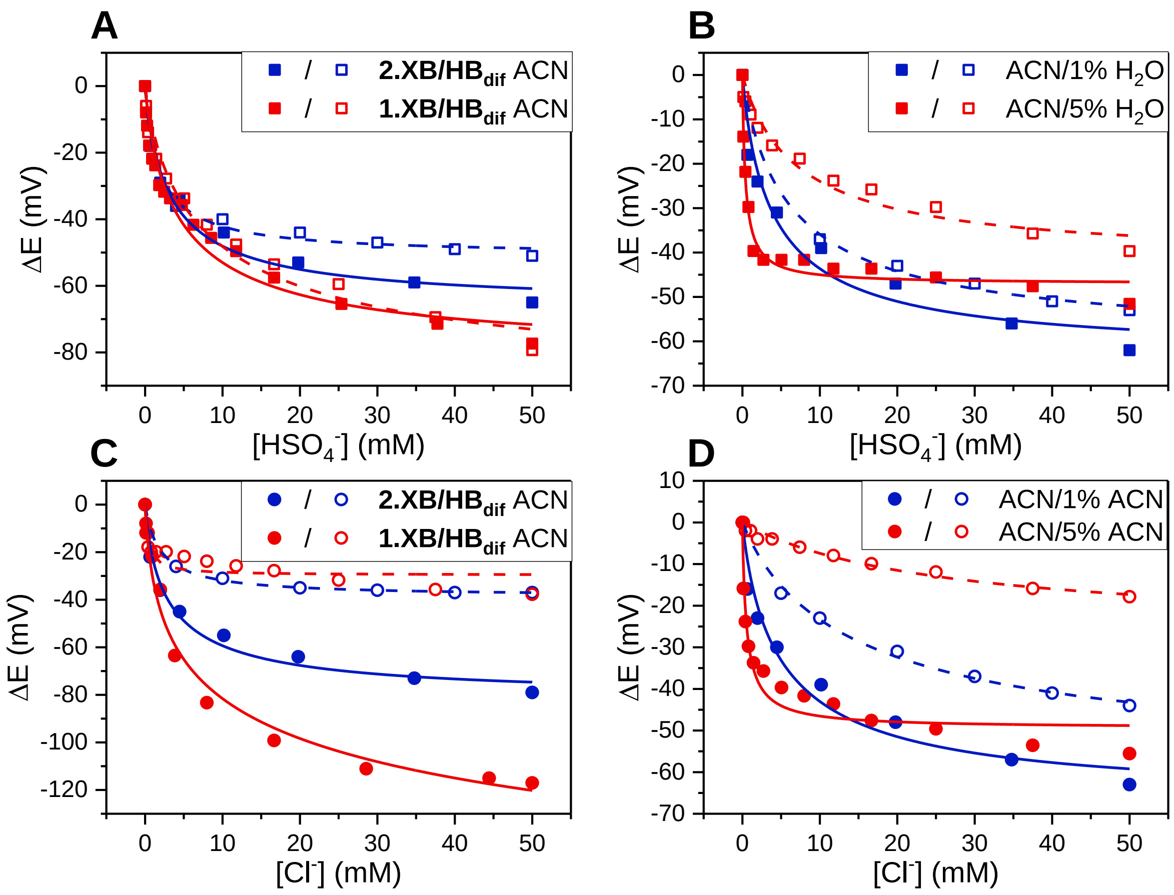

Figure S18. Comparison of cathodic voltammetric shifts of **1.XB/HB$_{dif}$** (red) and a similar Fc-isophthalamide receptor (structure above, Figure S17, denoted **2.XB/HB$_{dif}$**, blue) upon titration of A) and B) HSO_4^- C) Cl^- and D) Br^-. Comparisons are shown here for both receptors in ACN, as well as studies in ACN/5% H_2O and ACN/1% H_2O with **1.XB/HB$_{dif}$** and **2.XB/HB$_{dif}$** respectively ([**1/2.XB/HB**] = 0.1 mM, 100 mM $TBAClO_4$)**.** Solid lines represent fits to a 1:1 host-guest Nernst model (eqn. 2). Note the different y-axis scaling for all graphs.

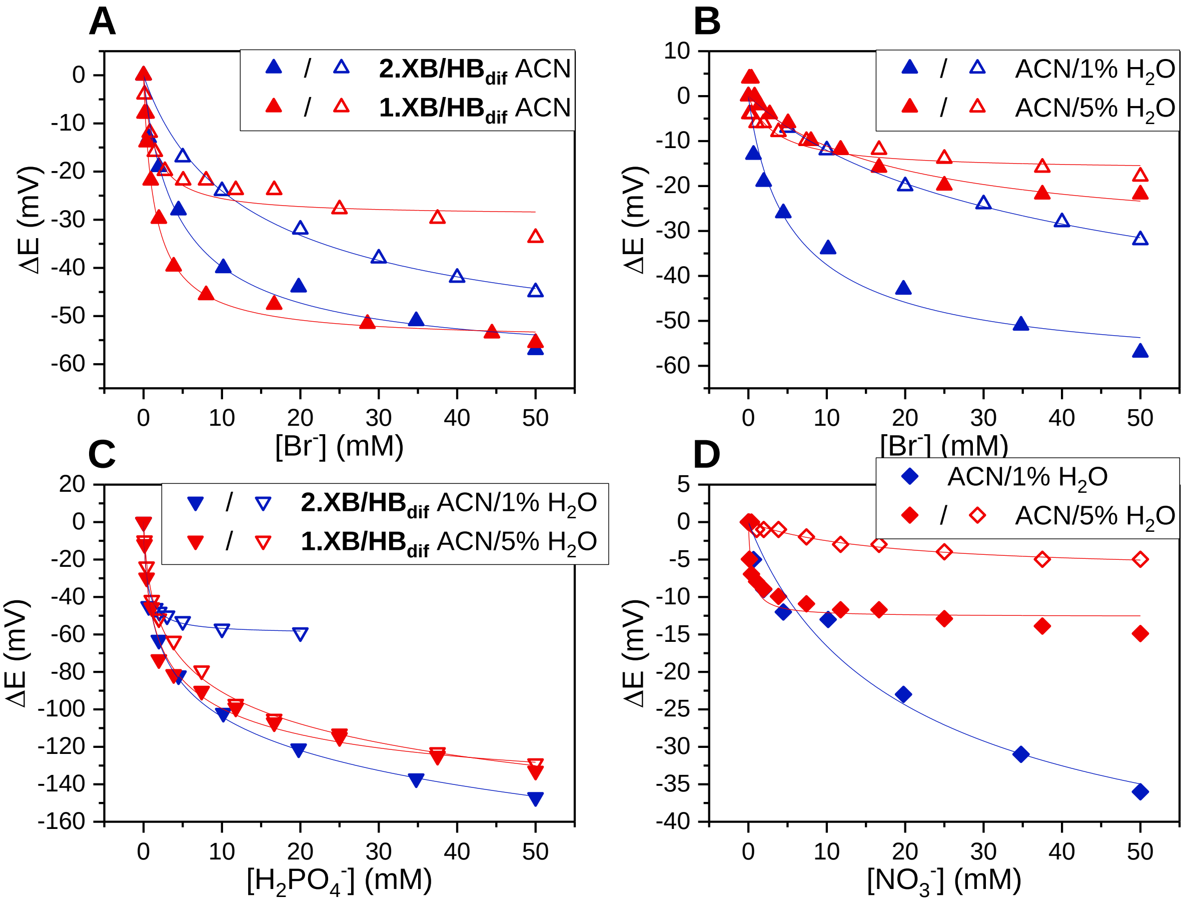

Figure S19. Comparison of cathodic voltammetric shifts of **1.XB/HB$_{dif}$** (red) and a similar Fc-isophthalamide receptor (structure above, Figure S17, denoted **2.XB/HB$_{dif}$**, blue) upon titration of A) and B) Br^- , C) $H_2PO_4^-$ and D) NO_3^-. Comparisons are made here for both receptors in ACN (where possible), as well as studies in ACN/5% H_2O and ACN/1% H_2O with **1.XB/HB$_{dif}$** and **2.XB/HB$_{dif}$** respectively ([**1/2.XB/HB**] = 0.1 mM, 100 mM $TBAClO_4$)**.** Solid lines represent fits to a 1:1 host-guest Nernst model (eqn. 2). Note the different y-axis scaling for all graphs.

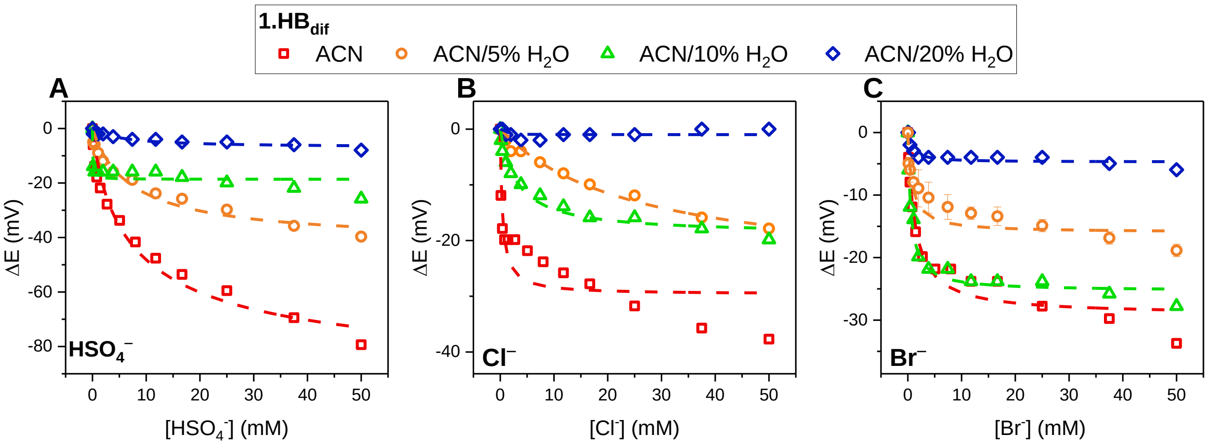

Figure S20. Cathodic voltammetric shifts of **1.HB$_{dif}$** in ACN with varying water content ([**1.HB**] = 0.1 mM with 100 mM TBAClO$_4$ supporting electrolyte), upon titration of A) HSO$_4^-$ B) Cl$^-$ and C) Br$^-$ fitted according to the 1:1 host-guest Nernst model (eqn. 2).

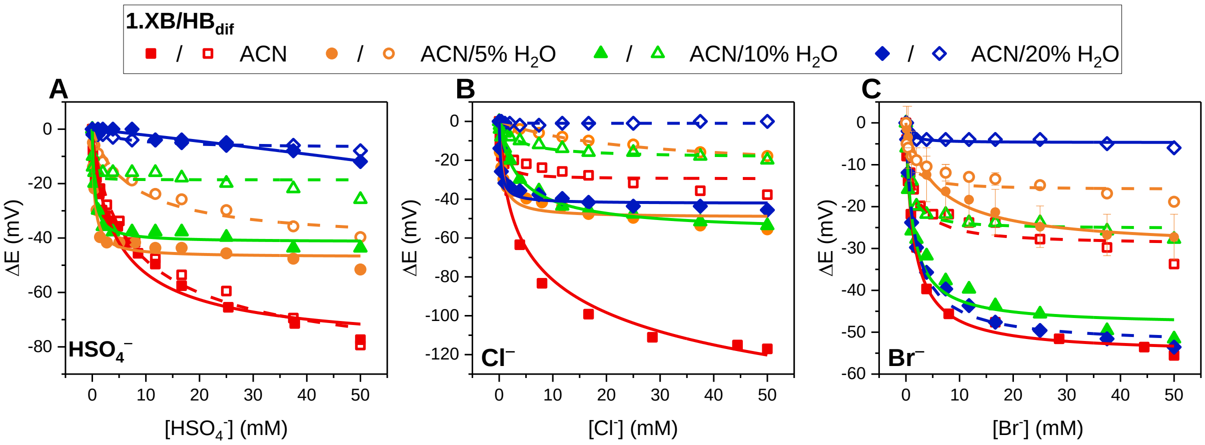

Figure S21. Cathodic voltammetric shifts of **1.XB$_{dif}$** (filled symbols) and **1.HB$_{dif}$** (empty symbols) in ACN with varying water content ([**1.XB/HB**] = 0.1 mM with 100 mM TBAClO$_4$ supporting electrolyte), upon titration of A) HSO$_4^-$ B) Cl$^-$ C) Br$^-$ fitted according to the 1:1 host-guest Nernst model (eqn. 2).

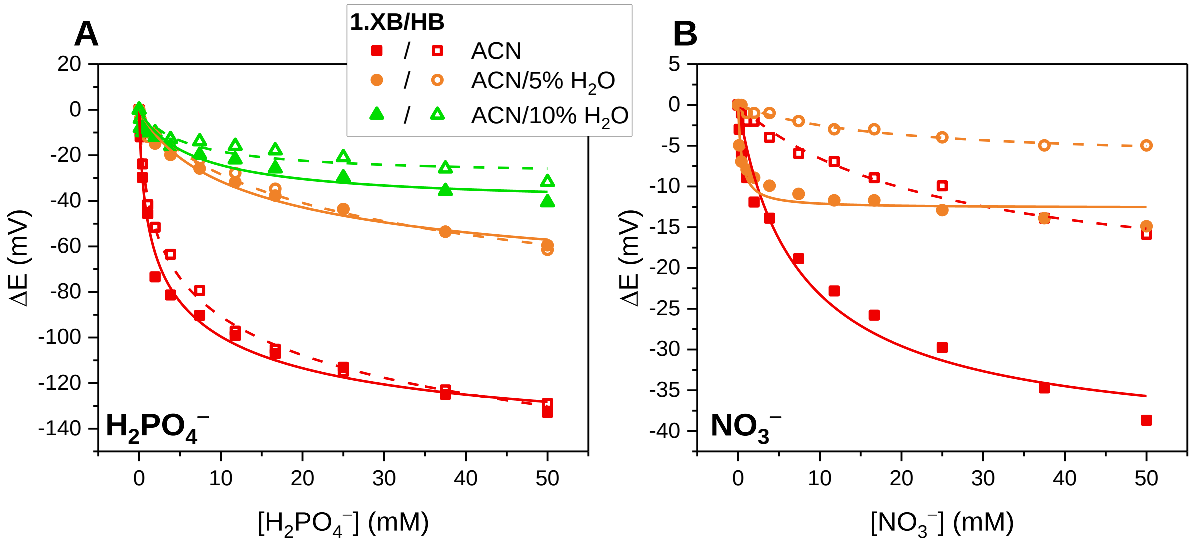

Figure S22. Cathodic voltammetric shifts of **1.XB$_{dif}$** (filled symbols) and **1.HB$_{dif}$** (empty symbols) in ACN with varying water content ([**1.XB/HB**] = 0.1 mM with 100 mM $TBAClO_4$ supporting electrolyte), upon titration of A) $H_2PO_4^-$ and B) NO_3^- fitted according to the 1:1 host-guest Nernst model (eqn. 2).

S4: Solvent Dependency of 1.XB/HB$_{SAM/dif}$

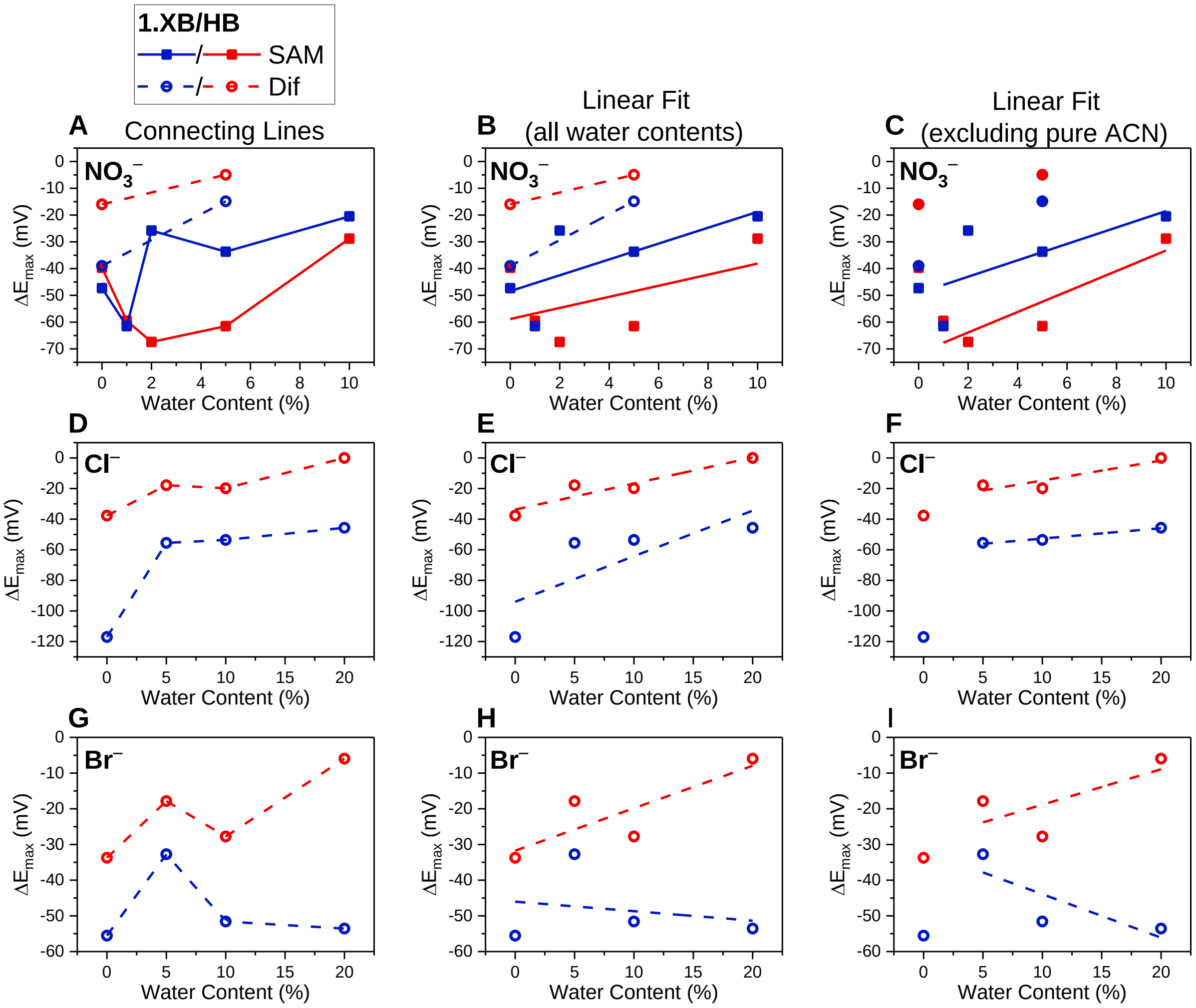

Figure S23. Maximum cathodic perturbation of $E_{1/2}$ (ΔE_{max}) of **1.XB/HB$_{SAM/dif}$** (blue and red symbols respectively for **1.XB/HB**, filled symbols represent the respective receptors as SAMs and empty symbols represent the receptors under diffusive conditions) in a range of solvent systems with varying water content, in the presence of 50 mM A), B) and C) NO_3^-, D), E) and F) Cl^- and G), H) and I) Br^-. A), D) and G) display connecting lines to guide the eye only, B), E) and H) show linear fits including data at all water contents and C), F) and I) show linear fits excluding data in pure ACN.

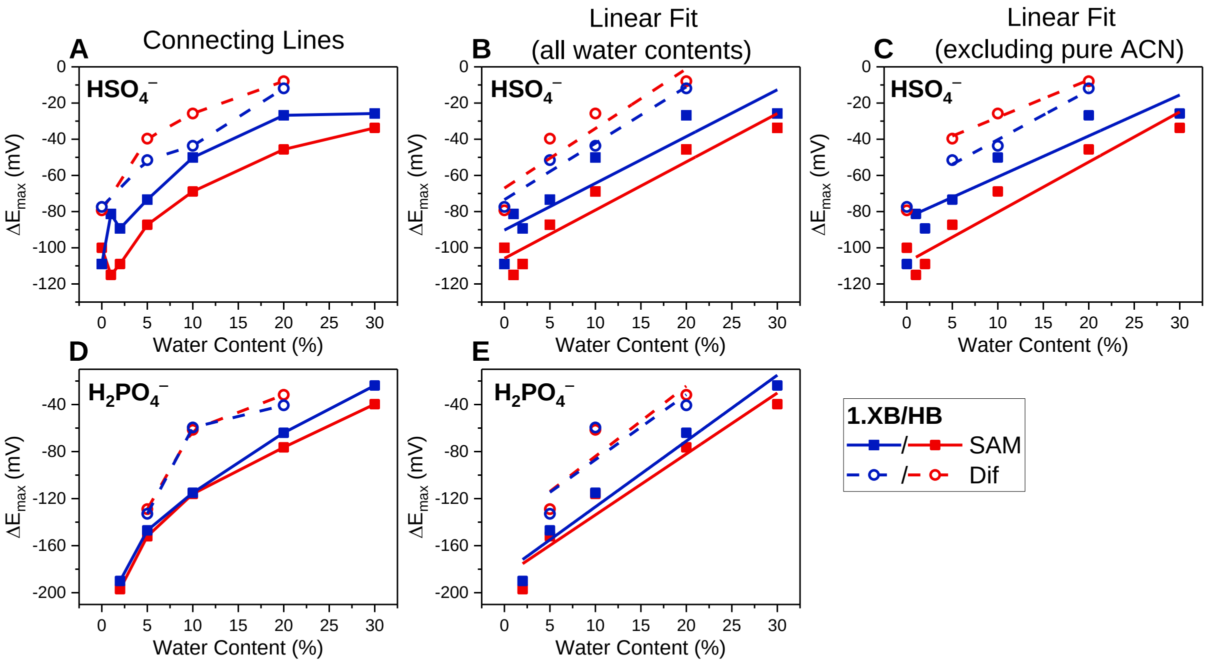

Figure S24. Maximum cathodic perturbation of $E_{1/2}$ (ΔE_{max}) of **1.XB/HB$_{SAM/dif}$** (blue and red symbols respectively for **1.XB/HB**, filled symbols represent the respective receptors as SAMs and empty symbols represent the receptors under diffusive conditions) in a range of solvent systems with varying water content, in the presence of 50 mM A), B) and C) HSO_4^- and D) and E) $H_2PO_4^-$. A) and D) display connecting lines to guide the eye only, B) and E) show linear fits including data at all water contents and C) shows linear fits excluding data in pure ACN.

Table S1. Tabulated values from linear fits shown in Figure S23 and S24 (slopes and R-square values) for plots of ΔE_{max} vs. water content from titrations of various anions with **1.XB/HB$_{SAM/dif}$** in all solvent systems. Values in parentheses denote the slopes excluding data points from measurements in ACN.

	Anion	Slope (mV/%H_2O)	R-square
**1.XB$_{dif}$**	HSO_4^-	3.1 ± 0.4 (2.7 ± 0.4)	0.97 (0.98)
**1.HB$_{dif}$**	HSO_4^-	3.3 ± 0.9 (2.1 ± 0.2)	0.86 (0.99)
**1.XB$_{SAM}$**	HSO_4^-	2.6 ± 0.5 (2.3 ± 0.4)	0.85 (0.88)
**1.HB$_{SAM}$**	HSO_4^-	2.7 ± 0.4 (2.8 ± 0.4)	0.92 (0.92)
**1.XB$_{dif}$**	NO_3^-	4.8	1
**1.HB$_{dif}$**	NO_3^-	2.2	1
**1.XB$_{SAM}$**	NO_3^-	3.0 ± 1.7 (3.1 ± 2.3)	0.51 (0.46)
**1.HB$_{SAM}$**	NO_3^-	2.1 ± 2.0 (3.8 ± 1.4)	0.26 (0.80)
**1.XB$_{dif}$**	$H_2PO_4^-$	5.5 ± 3.2	0.75
**1.HB$_{dif}$**	$H_2PO_4^-$	6.0 ± 2.6	0.84
**1.XB$_{SAM}$**	$H_2PO_4^-$	5.6 ± 0.6	0.96
**1.HB$_{SAM}$**	$H_2PO_4^-$	5.2 ± 0.8	0.94
**1.XB$_{dif}$**	Cl^-	3.0 ± 1.7 (0.7 ± 0.1)	0.59 (0.98)
**1.HB$_{dif}$**	Cl^-	1.7 ± 0.4 (1.3 ± 0.6)	0.89 (0.83)
**1.XB$_{dif}$**	Br^-	-0.3 ± 0.9 (-1.2 ± 0.9)	0.04 (0.66)
**1.HB$_{dif}$**	Br^-	1.2 ± 0.6 (1.0 ± 1.0)	0.70 (0.48)

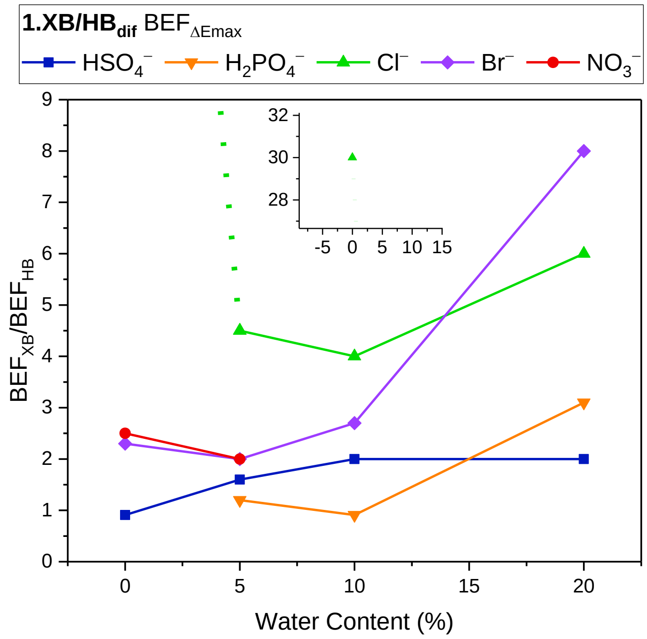

Figure S25. Binding enhancement factors (BEFs) of **1.XB/HB$_{dif}$** determined via eqn. 1 and the maximum voltammetric shift ΔE_{max} in response to 50 mM of various anions.

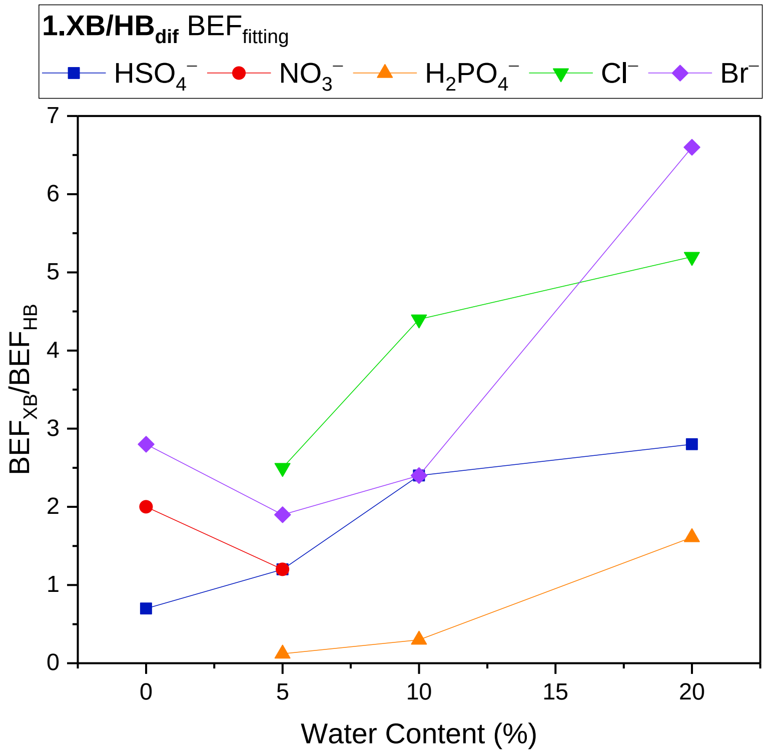

Figure S26. Binding enhancement factors (BEFs) of **1.XB/HB$_{dif}$** determined by isotherm fitting according to the 1:1 host-guest Nernst model (eqn 2). BEF_{XB}/BEF_{HB} for Cl^- in ACN is 410 (omitted for scaling reasons).

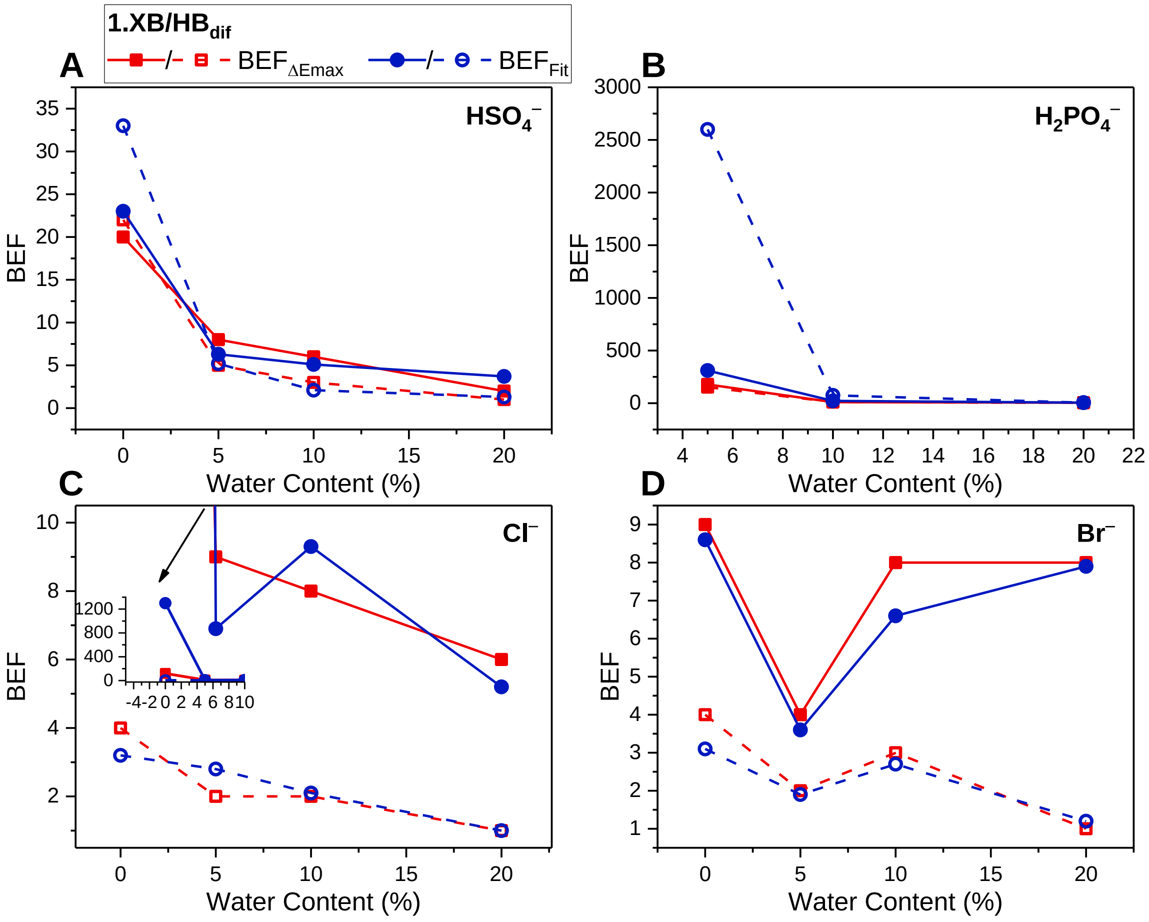

Figure S27. Comparison of binding enhancement factors (BEFs) of **1.XB/HB$_{dif}$** determined either by 1:1 host-guest Nernst model (eqn. 2; blue symbols) or the maximum shift in response to 50 mM of A) HSO_4^- B) $H_2PO_4^-$ C) Cl^- and D) Br^- (eqn. 1; red symbols).

S5: Interfacial Electrochemical Anion Sensing

S5.1 Surface Characterisation:

Attenuated total reflectance-Fourier transform infrared spectroscopy was performed with the free receptors **1.XB** or **1.HB** as well as **1.XB/HB$_{SAM}$** on planar gold substrates. Both procedures yielded very similar spectra (Figure S28), indicating successful surface immobilisation of **1.XB/HB** on Au and chemically similar interfaces between the XB and HB analogues. Asymmetric and symmetric methylene stretches at 2929 and 2856 cm^{-1}, respectively were consistent with previous reports for alkyl-thiol based SAMs.[4] The broad peaks at 1469 and 1741 cm^{-1} were attributed to the triazole[5] and ester groups, respectively. The prominent peak at 2355 cm^{-1} is attributed to atmospheric CO_2. A distinguishable peak which can be attributed to the C-I bonds in **1.XB/1.XB$_{SAM}$** could not be identified and is therefore expected to fall in the fingerprint region.

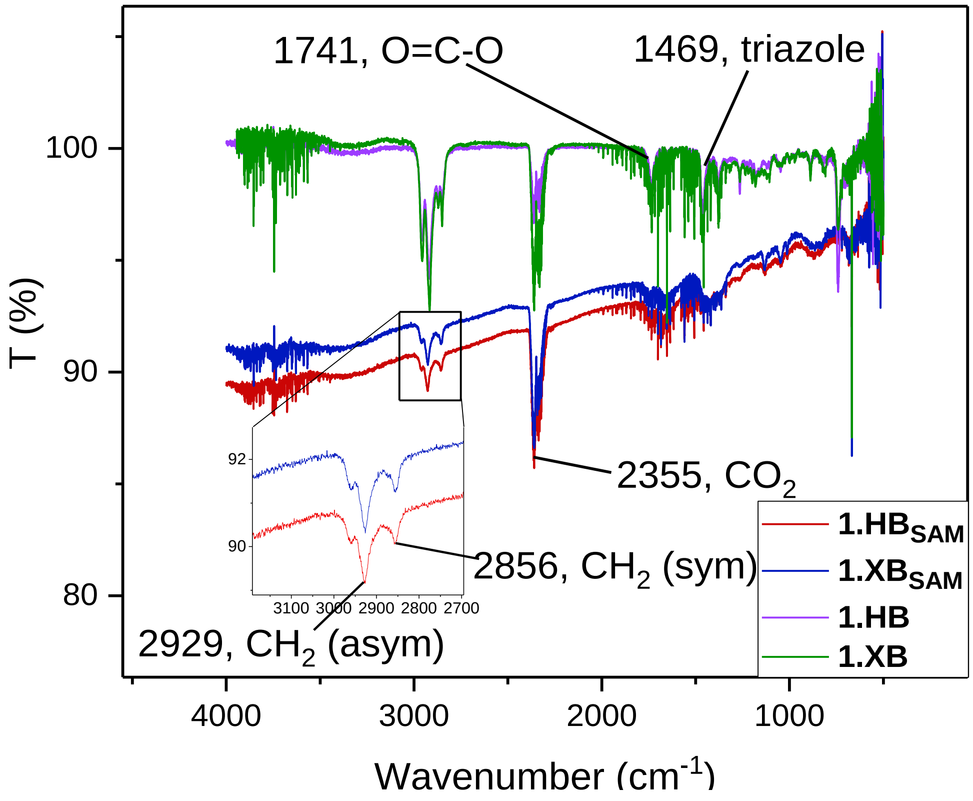

Figure S28. ATR-FTIR spectra of **1.XB/HB** and **1.XB/HB$_{SAM}$** on Au.

The significant difference in film dielectric is neither expected, nor easy to rationalise. XB motifs are generally associated with a higher hydrophobicity (and lower dielectric) as previously noted in similar XB/HB receptive films. We believe that the opposite trends reported herein (in terms of both film hydrophobicity as resolved by water contact angle measurements as well as the lower dielectric, Table 2) are not reflective of the receptor chemistry itself but instead arise from other factors, such as different interfacial receptor conformations (as supported by moderate discrepancies in receptor surface coverage and film thickness, Table 2) or differences in receptor hydration (as suggested from the lower water contact angle of $\mathbf{1.XB_{SAM}}$, indicating an increased binding site hydration).

S5.2 Electrochemical Surface Characterisation:

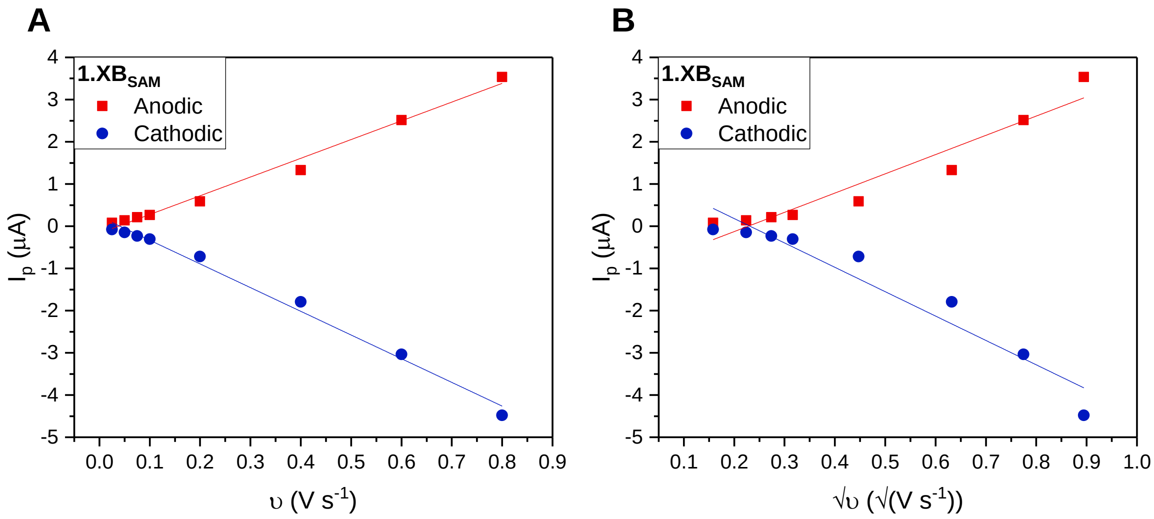

Figure S29. Peak current *vs.* A) scan rate B) the square-root of the scan rate with linear fits of **1.XB$_{SAM}$** in ACN (100 mM TBAClO$_4$).

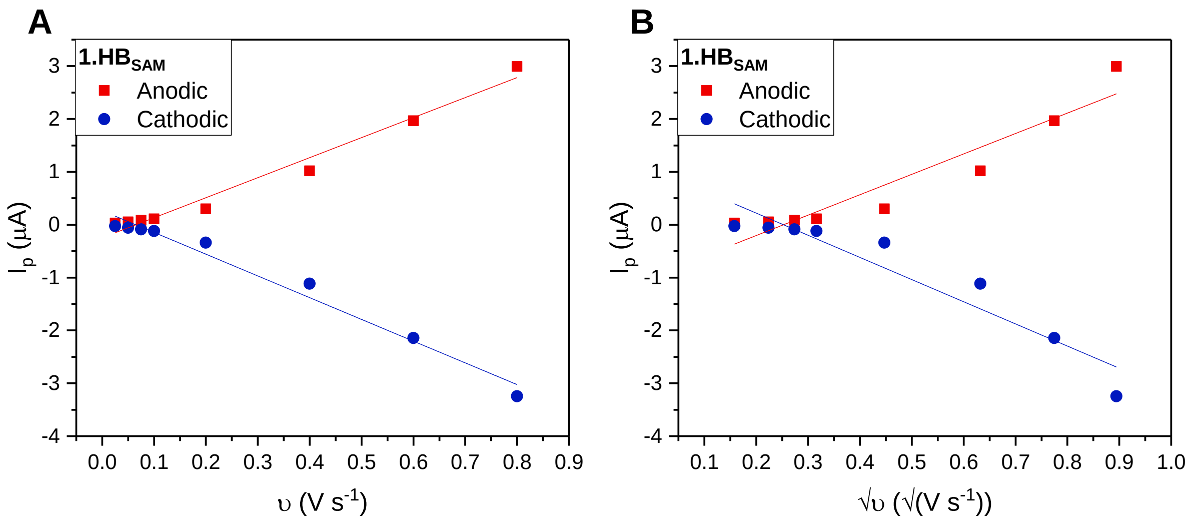

Figure S30. Peak current *vs.* A) scan rate B) the square-root of the scan rate with linear fits of **1.HB$_{SAM}$** in ACN (100 mM TBAClO$_4$).

S5.3 Anion Sensing Studies with 1.XB/HB$_{SAM}$

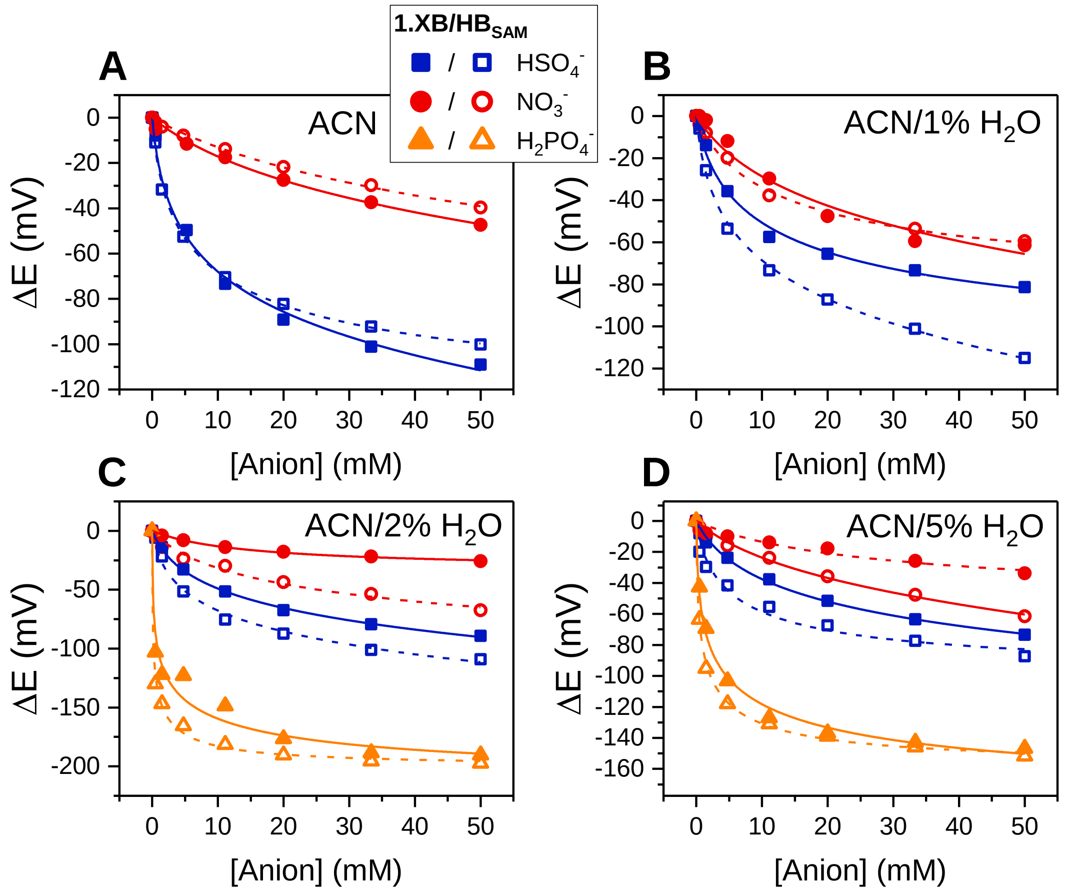

Figure S31. Cathodic voltammetric shifts of **1.XB/HB$_{SAM}$** upon titration of HSO_4^-, NO_3^-, $H_2PO_4^-$ in A) ACN B) ACN/1% H_2O C) ACN/2% H_2O and D) ACN/5% H_2O fitted according to the 1:1 host-guest Nernst model (eqn. 2).

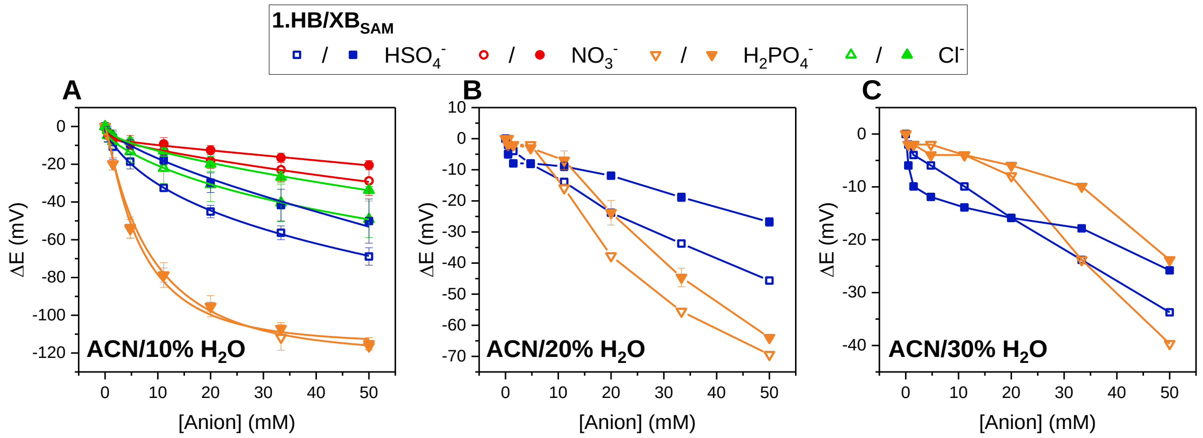

Figure S32. Cathodic voltammetric shifts of **1.XB/HB$_{SAM}$** upon titration of HSO_4^- and $H_2PO_4^-$ in A) ACN/10% H_2O fitted according to the 1:1 host-guest Nernst model (eqn. 2), and B) ACN/20% H_2O and C) ACN/30% H_2O with connecting lines shown to guide the eye only.

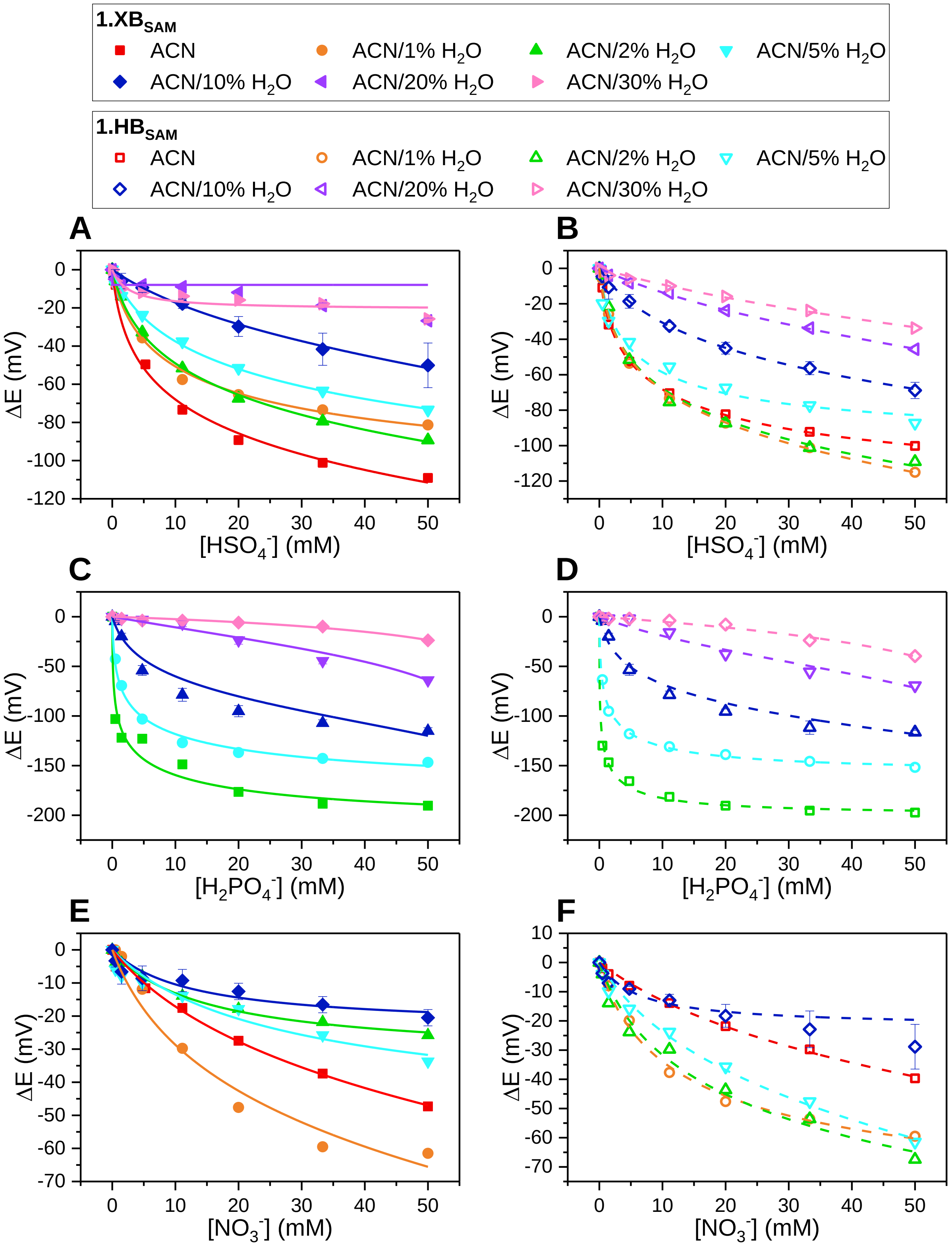

Figure S33. Cathodic voltammetric shifts of **1.XB**$_{SAM}$ upon titration of A) HSO_4^- C) $H_2PO_4^-$ and E) NO_3^- and the analogous response of **1.HB**$_{SAM}$ to B) HSO_4^- D) $H_2PO_4^-$ and F) NO_3^- fitted according to the 1:1 host-guest Nernst model (eqn. 2).

S6: Maximum voltammetric shifts, BEF ($BEF_{\Delta Emax}$ and BEF_{Fit}) and SEF data

S6.1 Maximum Cathodic Shifts for $1.XB/HB_{dif/SAM}$

Table S2. Maximum cathodic voltammetric shift (ΔE_{max}) of $\mathbf{1.XB/HB_{SAM/dif}}$ in response to 50 mM of various anions in a range of ACN/H_2O solvent systems.

	ΔE_{max} (mV)			
	$1.XB_{dif}$	$1.HB_{dif}$	$1.XB_{SAM}$	$1.HB_{SAM}$
ACN	Cl^- > HSO_4^- > Br^- > NO_3^- 120 > 77 > 56 > 39	HSO_4^- > Cl^- > Br^- > NO_3^- 79 > 38 > 34 > 16	HSO_4^- > NO_3^- 110 > 47	HSO_4^- > NO_3^- 100 > 40
ACN/1% H_2O	/	/	HSO_4^- > NO_3^- 81 > 62	HSO_4^- > NO_3^- 120 > 60
ACN/2% H_2O	/	/	$H_2PO_4^-$ > HSO_4^- > NO_3^- 190 > 89 > 26	$H_2PO_4^-$ > HSO_4^- > NO_3^- 200 > 110 > 67
ACN/5% H_2O	$H_2PO_4^-$ > Cl^- > HSO_4^- > Br^- > NO_3^- 133 > 56 > 52 > 33 > 15	$H_2PO_4^-$ > HSO_4^- > Cl^- = Br^- > NO_3^- 129 > 40 > 18 = 18 > 5	$H_2PO_4^-$ > HSO_4^- > NO_3^- 150 > 73 > 34	$H_2PO_4^-$ > HSO_4^- > NO_3^- 150 > 87 > 62
ACN/10% H_2O	$H_2PO_4^-$ > Cl^- > Br^- >HSO_4^- 60 > 54 > 52 > 44	$H_2PO_4^-$ > Br^- > HSO_4^- > Cl^- 62 > 28 > 26 > 20	$H_2PO_4^-$ > HSO_4^- > NO_3^- 120 > 50 > 21	$H_2PO_4^-$ > HSO_4^- > NO_3^- 120 > 69 > 29
ACN/20% H_2O	Br^- > Cl^- > $H_2PO_4^-$ >HSO_4^- 54 > 46 > 41 > 12	$H_2PO_4^-$ > HSO_4^- > Br^- > Cl^- 32 > 8 > 6 > 0	$H_2PO_4^-$ > HSO_4^- 64 > 27	$H_2PO_4^-$ > HSO_4^- 76 > 46
ACN/30% H_2O	/	/	HSO_4^- > $H_2PO_4^-$ 26 > 24	$H_2PO_4^-$ > HSO_4^- 40 > 34

S6.2 Binding Enhancement Factors (BEFs) from ΔE_{max} and 1:1 Host-guest Nernst Model for 1.XB/HB$_{dif}$

Table S3. Diffusive binding constants of the oxidised (K_{Ox}) and neutral (K_{red}) receptors (M^{-1}), BEFs (K_{Ox}/K_{red}) and XB/HB BEF ratios with HSO_4^- determined from fitting isotherms with a 1:1 host-guest Nernst model (eqn. 2) or the maximum shift (eqn. 1) in response to 50 mM [X^-].

Diffusive	Solvent System	K_{Ox}	K_{Red}	BEF_{Fit}	$(BEF_{XB}/BEF_{HB})_{Fit}$	$BEF_{\Delta Emax}$	$(BEF_{XB}/BEF_{HB})_{\Delta Emax}$
**1.XB$_{dif}$**	ACN	1050 ± 128	45.2 ± 13.0	23	0.70	20	0.91
**1.HB$_{dif}$**	ACN	697 ± 107	21.4 ± 9.85	33		22	
**1.XB$_{dif}$**	ACN/5% H_2O	6290 ± 849	1000 ± 159	6.3	1.2	8	1.6
**1.HB$_{dif}$**	ACN/5% H_2O	307 ± 65.3	59.6 ± 19.9	5.2		5	
**1.XB$_{dif}$**	ACN/10% H_2O	5410 ± 675	1070 ± 153	5.1	2.4	6	2
**1.HB$_{dif}$**	ACN/10% H_2O	23500 ± 17000	11300 ± 8420	2.1		3	
**1.XB$_{dif}$**	ACN/20% H_2O	4840 ± 1520	1320 ± 459	3.7	2.8	2	2
**1.HB$_{dif}$**	ACN/20% H_2O	219 ± 10100	166 ± 82.2	1.3		1	

Table S4. Diffusive binding constants of the oxidised (K_{Ox}) and neutral (K_{red}) receptors (M^{-1}), BEFs (K_{Ox}/K_{red}) and XB/HB BEF ratios with NO_3^- determined from fitting isotherms with a 1:1 host-guest Nernst model (eqn. 2) or the maximum shift (eqn. 1) in response to 50 mM [X^-].

Diffusive	Solvent System	K_{Ox}	K_{Red}	BEF_{Fit}	$(BEF_{XB}/BEF_{HB})_{Fit}$	$BEF_{\Delta Emax}$	$(BEF_{XB}/BEF_{HB})_{\Delta Emax}$
**1.XB$_{dif}$**	ACN	286 ± 54.5	56.0 ± 16.9	5.1	2.0	5	2.5
**1.HB$_{dif}$**	ACN	61.5 ± 10.6	25.1 ± 6.55	2.5		2	
**1.XB$_{dif}$**	ACN/5% H_2O	2930 ± 919	1790 ± 584	1.6	1.2	2	2
**1.HB$_{dif}$**	ACN/5% H_2O	65.5 ± 12.9	50.1 ± 11.0	1.3		1	

Table S5. Diffusive binding constants of the oxidised (K_{Ox}) and neutral (K_{red}) receptors (M^{-1}), BEFs (K_{Ox}/K_{red}) and XB/HB BEF ratios with $H_2PO_4^-$ determined from fitting isotherms with a 1:1 host-guest Nernst model (eqn. 2) or the maximum shift (eqn. 1) in response to 50 mM [X^-].

Diffusive	Solvent System	K_{Ox}	K_{Red}	BEF_{Fit}	$(BEF_{XB}/BEF_{HB})_{Fit}$	$BEF_{\Delta Emax}$	$(BEF_{XB}/BEF_{HB})_{\Delta Emax}$
**1.XB$_{dif}$**	ACN/5% H_2O	5640 ± 546	18.0 ± 6.82	310	0.12	178	1.2
**1.HB$_{dif}$**	ACN/5% H_2O	3390 ± 166	1.28 ± 2.11	2600		152	
**1.XB$_{dif}$**	ACN/10% H_2O	285 ± 39.0	12.9 ± 6.20	22	0.30	10	0.91
**1.HB$_{dif}$**	ACN/10% H_2O	211 ± 38.6	2.87 ± 5.83	74		11	
**1.XB$_{dif}$**	ACN/20% H_2O	323 ± 102	64.0 ± 31.1	5.0	1.61	5	1.7
**1.HB$_{dif}$**	ACN/20% H_2O	310 ± 123	100 ± 52.2	3.1		3	

Table S6. Diffusive binding constants of the oxidised (K_{Ox}) and neutral (K_{red}) receptors (M^{-1}), BEFs (K_{Ox}/K_{red}) and XB/HB BEF ratios with Cl^- determined from fitting isotherms with a 1:1 host-guest Nernst model (eqn. 2) or the maximum shift (eqn. 1) in response to 50 mM [X^-].

Diffusive	Solvent System	K_{Ox}	K_{Red}	BEF_{Fit}	$(BEF_{XB}/BEF_{HB})_{Fit}$	$BEF_{\Delta Emax}$	$(BEF_{XB}/BEF_{HB})_{\Delta Emax}$
**1.XB$_{dif}$**	ACN	2360 ± 308	1.80 ± 4.95	1300	410	120	30
**1.HB$_{dif}$**	ACN	4290 ± 1820	1350 ± 633	3.2		4	
**1.XB$_{dif}$**	ACN/5% H_2O	4830 ± 10.3	702 ± 180	6.9	2.5	9	4.5
**1.HB$_{dif}$**	ACN/5% H_2O	63.9 ± 12.2	22.7 ± 7.10	2.8		2	
**1.XB$_{dif}$**	ACN/10% H_2O	822 ± 44.2	88.0 ± 7.47	9.3	4.4	8	4.0
**1.HB$_{dif}$**	ACN/10% H_2O	468 ± 85.1	224 ± 46.5	2.1		2	
**1.XB$_{dif}$**	ACN/20% H_2O	7340 ± 1140	1410 ± 247	5.2	5.2	6	6.0
**1.HB$_{dif}$**	ACN/20% H_2O	2790 ± 5570	2690 ± 5370	1.0		1	

Table S7. Diffusive binding constants of the oxidised (K_{Ox}) and neutral (K_{red}) receptors (M^{-1}), BEFs (K_{Ox}/K_{red}) and XB/HB BEF ratios with Br^- determined from fitting isotherms with a 1:1 host-guest Nernst model (eqn. 2) or the maximum shift (eqn. 1) in response to 50 mM [X^-].

Diffusive	Solvent System	K_{Ox}	K_{Red}	BEF_{Fit}	$(BEF_{XB}/BEF_{HB})_{Fit}$	$BEF_{\Delta Emax}$	$(BEF_{XB}/BEF_{HB})_{\Delta Emax}$
**1.XB$_{dif}$**	ACN	2010 ± 153	233 ± 23.6	8.6	2.8	9	2.3
**1.HB$_{dif}$**	ACN	1320 ± 266	421 ± 99.4	3.1		4	
**1.XB$_{dif}$**	ACN/5% H_2O	94.6 ± 24.8	26.1 ± 11.9	3.6	1.9	4	2.0
**1.HB$_{dif}$**	ACN/5% H_2O	393 ± 126	206 ± 74.6	1.9		2	
**1.XB$_{dif}$**	ACN/10% H_2O	2030 ± 349	307 ± 67.2	6.6	2.4	8	2.7
**1.HB$_{dif}$**	ACN/10% H_2O	2780 ± 448	1030 ± 182	2.7		3	
**1.XB$_{dif}$**	ACN/20% H_2O	1740 ± 151	220 ± 25.5	7.9	6.6	8	8.0
**1.HB$_{dif}$**	ACN/20% H_2O	1590 ± 579	1320 ± 490	1.2		1	

S6.3 Binding Enhancement Factors (BEFs) from ΔE_{max} and 1:1 Host-guest Nernst Model for 1.XB/HB$_{SAM}$

Table S8. Interfacial binding constants of the oxidised (K_{Ox}) and neutral (K_{red}) receptors (M^{-1}), BEFs (K_{Ox}/K_{red}) and XB/HB BEF ratios with HSO_4^- determined from (1) fitting isotherms with a 1:1 host-guest Nernst model (eqn. 2) and (2) the maximum shift (eqn. 1) in response to 50 mM [X^-].

SAMs	Solvent System	K_{Ox}	K_{Red}	BEF_{Fit}	$(BEF_{XB}/BEF_{HB})_{Fit}$	$BEF_{\Delta Emax}$	$(BEF_{XB}/BEF_{HB})_{\Delta Emax}$
**1.XB$_{SAM}$**	ACN	1270 ± 139	-3.36 ± 3.13	//	/	73	1.5
**1.HB$_{SAM}$**	ACN	1490 ± 77.4	10.9 ± 2.61	140		49	
**1.XB$_{SAM}$**	ACN/1% H_2O	688 ± 76.2	8.99 ± 4.89	77	/	23	0.21
**1.HB$_{SAM}$**	ACN/1% H_2O	1280 ± 111	-5.50 ± 2.31	//		110	
**1.XB$_{SAM}$**	ACN/2% H_2O	559 ± 22.6	-2.86 ± 1.13	//	/	32	0.44
**1.HB$_{SAM}$**	ACN/2% H_2O	1260 ± 170	-3.61 ± 3.99	//		73	
**1.XB$_{SAM}$**	ACN/5% H_2O	336 ± 34.1	0.704 ± 3.07	480	11	17	0.57
**1.HB$_{SAM}$**	ACN/5% H_2O	1140 ± 230	25.9 ± 13.7	44		30	
**1.XB$_{SAM}$**	ACN/10% H_2O	83.8 ± 10.5	-6.13 ± 3.37	//	/	7	0.47
**1.HB$_{SAM}$**	ACN/10% H_2O	220 ± 9.63	-3.16 ± 1.31	//		15	
**1.XB$_{SAM}$**	ACN/20% H_2O					3	0.5
**1.HB$_{SAM}$**	ACN/20% H_2O					6	
**1.XB$_{SAM}$**	ACN/30% H_2O					3	0.75
**1.HB$_{SAM}$**	ACN/30% H_2O					4	

/-Meaningless $(BEF_{XB}/BEF_{HB})_{Fit}$ due to nonsensical BEF_{Fit} values (e.g. negative K_{red}, large errors). //- Meaningless BEF_{Fit} due to unrealistic values for K_{Ox} and/or K_{red} by 1:1 host-guest Nernst equation (eqn. 2) without value limitations or when K_{red} was restricted to >0. Empty cells represent isotherms which could not be fitted to 1:1 host-guest Nernst equation (eqn. 2).

Table S9. Interfacial binding constants of the oxidised (K_{Ox}) and neutral (K_{red}) receptors (M^{-1}), BEFs (K_{Ox}/K_{red}) and XB/HB BEF ratios with NO_3^- determined from (1) fitting isotherms with a 1:1 host-guest Nernst model (eqn. 2) and (2) the maximum shift in response to 50 mM [X^-].

SAMs	Solvent System	K_{Ox}	K_{Red}	BEF_{Fit}	$(BEF_{XB}/BEF_{HB})_{Fit}$	$BEF_{\Delta Emax}$	$(BEF_{XB}/BEF_{HB})_{\Delta Emax}$
**1.XB**$_{SAM}$	ACN	90.4 ± 11.7	-2.36 ± 2.56	//	/	6	1.2
**1.HB**$_{SAM}$	ACN	63.2 ± 6.40	-1.89 ± 1.81	//		5	
**1.XB**$_{SAM}$	ACN/1% H_2O	197 ± 43.6	-3.24 ± 5.12	//	/	11	1.1
**1.HB**$_{SAM}$	ACN/1% H_2O	323 ± 29.1	12.6 ± 3.94	26		10	
**1.XB**$_{SAM}$	ACN/2% H_2O	125 ± 23.5	34.6 ± 10.4	3.6	0.02	3	0.21
**1.HB**$_{SAM}$	ACN/2% H_2O	249 ± 44.8	1.39 ± 5.32	179		14	
**1.XB**$_{SAM}$	ACN/5% H_2O	140 ± 20.1	-4.82 ± 2.83	//	/	4	0.36
**1.HB**$_{SAM}$	ACN/5% H_2O	88.9 ± 35.6	11.5 ± 12.7	7.7		11	
**1.XB**$_{SAM}$	ACN/10% H_2O	124 ± 64.6	49.3 ± 34.0	2.5	1.0	2	0.67
**1.HB**$_{SAM}$	ACN/10% H_2O	251 ± 104	106 ± 66.2	2.4		3	

/-Meaningless $(BEF_{XB}/BEF_{HB})_{Fit}$ due to nonsensical BEF_{Fit} values (e.g. negative K_{red}, large errors). //- Meaningless BEF_{Fit} due to unrealistic values for K_{Ox} and/or K_{red} by 1:1 host-guest Nernst equation (eqn. 2) without value limitations or when K_{red} was restricted to >0. Empty cells represent isotherms which could not be fitted to 1:1 host-guest Nernst equation (eqn. 2).

Table S10. Interfacial binding constants of the oxidised (K_{Ox}) and neutral (K_{red}) receptors (M^{-1}), BEFs (K_{Ox}/K_{red}) and XB/HB BEF ratios with $H_2PO_4^-$ determined from (1) fitting isotherms with a 1:1 host-guest Nernst model (eqn. 2) and (2) the maximum shift in response to 50 mM [X^-].

SAMs	Solvent System	K_{Ox}	K_{Red}	BEF_{Fit}	$(BEF_{XB}/BEF_{HB})_{Fit}$	$BEF_{\Delta Emax}$	$(BEF_{XB}/BEF_{HB})_{\Delta Emax}$
**$1.XB_{SAM}$**	ACN/2% H_2O	58900 ± 16900	16.6 ± 19.2	3500	1.5	1600	0.67
**$1.HB_{SAM}$**	ACN/2% H_2O	259000 ± 30600	106 ± 22.3	2400		2400	
**$1.XB_{SAM}$**	ACN/5% H_2O	11200 ± 1170	11.8 ± 6.06	950	2.1	340	1
**$1.HB_{SAM}$**	ACN/5% H_2O	25300 ± 1620	54.0 ± 7.57	470		340	
**$1.XB_{SAM}$**	ACN/10% H_2O	816 ± 205	-12.3 ± 3.52	//	/	110	1
**$1.HB_{SAM}$**	ACN/10% H_2O	1210 ± 286	-7.86 ± 4.09	//		110	
**$1.XB_{SAM}$**	ACN/20% H_2O					12	0.6
**$1.HB_{SAM}$**	ACN/20% H_2O					19	
**$1.XB_{SAM}$**	ACN/30% H_2O					3	0.6
**$1.HB_{SAM}$**	ACN/30% H_2O					5	

/-Meaningless $(BEF_{XB}/BEF_{HB})_{Fit}$ due to nonsensical BEF_{Fit} values (e.g. negative K_{red}, large errors). //- Meaningless BEF_{Fit} due to unrealistic values for K_{Ox} and/or K_{red} by 1:1 host-guest Nernst equation (eqn. 2) without value limitations or when K_{red} was restricted to >0. Empty cells represent isotherms which could not be fitted to 1:1 host-guest Nernst equation (eqn. 2).

S6.4 Surface Enhancement Factors (SEFs) for 1.XB/$HB_{dif/SAM}$

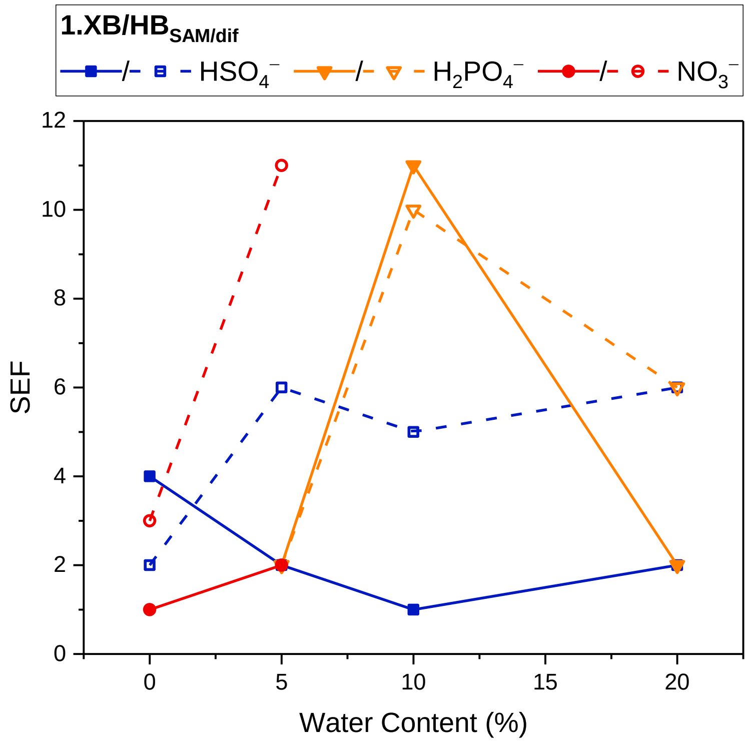

Figure S34. Surface enhancement factor (SEF = BEF_{SAM}/BEF_{dif}) for **1.XB/HB_{dif}** in response to the oxoanions (HSO_4^-, $H_2PO_4^-$ and NO_3^-) in a range of organic/aqueous solvent systems up to ACN/20% H_2O.

Table S11. Surface enhancement factor (BEF_{SAM}/BEF_{dif}) data calculated from BEFs from maximum cathodic shifts (ΔE_{max}) in response to HSO_4^-.

HSO_4^-	Solvent System	BEF_{SAM}	BEF_{dif}	SEF (BEF_{SAM}/BEF_{dif})
**1.XB**	ACN	73	20	4
**1.HB**	ACN	49	22	2
**1.XB**	ACN/5% H_2O	17	8	2
**1.HB**	ACN/5% H_2O	30	5	6
**1.XB**	ACN/10% H_2O	7	6	1
**1.HB**	ACN/10% H_2O	15	3	5
**1.XB**	ACN/20% H_2O	3	2	2
**1.HB**	ACN/20% H_2O	6	1	6

Table S12. Surface enhancement factor (BEF_{SAM}/BEF_{dif}) data calculated from BEFs from maximum cathodic shifts (ΔE_{max}) in response to NO_3^-.

NO_3^-	Solvent System	BEF_{SAM}	BEF_{dif}	SEF (BEF_{SAM}/BEF_{dif})
**1.XB**	ACN	6	5	1
**1.HB**	ACN	5	2	3
**1.XB**	ACN/5% H_2O	4	2	2
**1.HB**	ACN/5% H_2O	11	1	11

Table S13. Surface enhancement factor (BEF_{SAM}/BEF_{dif}) data calculated from BEFs from maximum cathodic shifts (ΔE_{max}) in response to $H_2PO_4^-$.

$H_2PO_4^-$	Solvent System	BEF_{SAM}	BEF_{dif}	SEF (BEF_{SAM}/BEF_{dif})
**1.XB**	ACN/5% H_2O	340	178	2
**1.HB**	ACN/5% H_2O	340	152	2
**1.XB**	ACN/10% H_2O	110	10	11
**1.HB**	ACN/10% H_2O	110	11	10
**1.XB**	ACN/20% H_2O	12	5	2
**1.HB**	ACN/20% H_2O	19	3	6

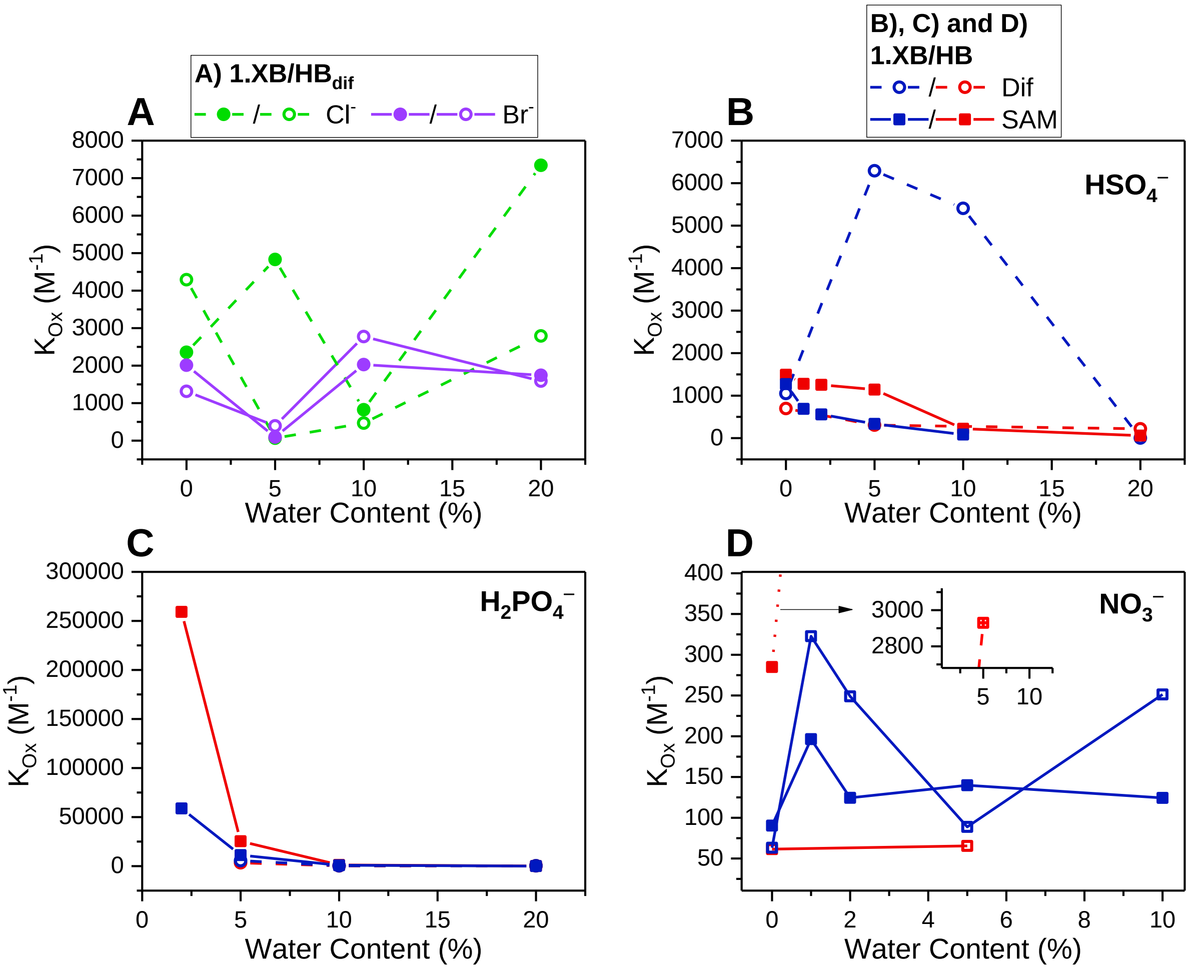

Figure S35. K_{ox} values determined from 1:1 host-guest Nernst fitting (eqn. 2) of isotherms vs water content of solvent system for **1.XB$_{dif}$** (filled symbols) and **1.HB$_{dif}$** (empty symbols) in response to A) Cl^- and Br^-, and a comparison of **1.XB/HB$_{SAM}$** (red) and **1.XB/HB$_{dif}$** (blue) for B) HSO_4^- C) $H_2PO_4^-$ and D) and NO_3^-.

References:

[1] R. Hein, A. Borissov, M. D. Smith, P. D. Beer, J. J. Davis, *Chem. Commun.* **2019**, *55*, 4849-4852.

[2] S.-H. Hwang, C. N. Moorefield, H.-C. Cha, P. Wang, G. R. Newkome, *Des. Monomers Polym.* **2006**, *9*, 413-424.

[3] R. Hein, X. Li, P. D. Beer, J. J. Davis, *Chem. Sci.* **2021**, *12*, 2433-2440.

[4] a) J. Lehr, J. R. Weeks, A. Santos, G. T. Feliciano, M. I. G. Nicholson, J. J. Davis, P. R. Bueno, *Phys. Chem. Chem. Phys.* **2017**, *19*, 15098-15109; b) M. D. Porter, T. B. Bright, D. L. Allara, C. E. D. Chidsey, *J. Am. Chem. Soc.* **1987**, *109*, 3559-3568.

[5] H. Li, Q. Zheng, C. Han, *Analyst* **2010**, *135*, 1360-1364.
